# The mechanism of the nucleo-sugar selection by multi-subunit RNA polymerases

**DOI:** 10.1038/s41467-021-21005-w

**Published:** 2021-02-04

**Authors:** Janne J. Mäkinen, Yeonoh Shin, Eeva Vieras, Pasi Virta, Mikko Metsä-Ketelä, Katsuhiko S. Murakami, Georgiy A. Belogurov

**Affiliations:** 1 Department of Biochemistry, University of Turku, Turku, Finland; 2 Department of Biochemistry and Molecular Biology, The Center for RNA Molecular Biology, The Pennsylvania State University, University Park, PA USA; 3 Department of Chemistry, University of Turku, Turku, Finland

**Keywords:** Enzyme mechanisms, Transcription, X-ray crystallography

## Abstract

RNA polymerases (RNAPs) synthesize RNA from NTPs, whereas DNA polymerases synthesize DNA from 2′dNTPs. DNA polymerases select against NTPs by using steric gates to exclude the 2′OH, but RNAPs have to employ alternative selection strategies. In single-subunit RNAPs, a conserved Tyr residue discriminates against 2′dNTPs, whereas selectivity mechanisms of multi-subunit RNAPs remain hitherto unknown. Here, we show that a conserved Arg residue uses a two-pronged strategy to select against 2′dNTPs in multi-subunit RNAPs. The conserved Arg interacts with the 2′OH group to promote NTP binding, but selectively inhibits incorporation of 2′dNTPs by interacting with their 3′OH group to favor the catalytically-inert 2′-endo conformation of the deoxyribose moiety. This deformative action is an elegant example of an active selection against a substrate that is a substructure of the correct substrate. Our findings provide important insights into the evolutionary origins of biopolymers and the design of selective inhibitors of viral RNAPs.

## Abstract (web summary)

RNA and DNA polymerases need to discriminate efficiently against closely related nucleotide triphosphate substrates. Here, the authors show that a conserved Arg residue is the major determinant of selectivity against deoxyribonucleoside substrates by multisubunit RNA polymerases.

## Introduction

**Fig. 1:**
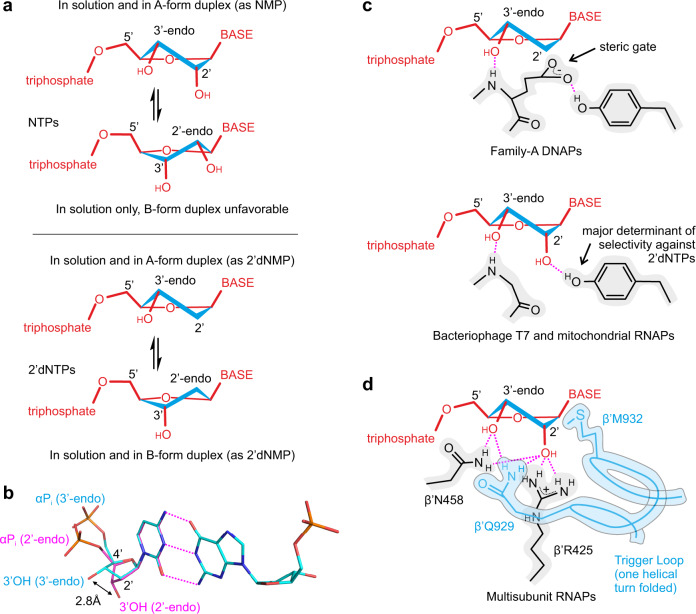
Recognition of NTPs and 2′dNTPs by nucleic acid polymerases. **a** NTPs and 2′dNTPs equilibrate freely between 3′-endo and 2′-endo conformations in solution. A- and B-form duplexes of RNA and DNA constrain sugar moieties of nucleotides in 3′-endo and 2′-endo conformations, respectively. C1′–C2′–C3′–C4′ bonds of the sugar moieties are colored cyan. **b** Watson–Crick base pairing of the 3′-endo (cyan) and 2′-endo (magenta) conformers to the template nucleic acid results in markedly different positions of the 3′OH group and the α-phosphate relative to the base-pairing plane. **c**, **d** DNAPs and RNAPs stabilize 3′-endo conformers of their cognate substrates. The illustrations reflect the crystallographically documented arrangement of the active site residues but are not accurate projections of 3D structures. Dashed magenta lines depict polar interactions and hydrogen bonds.

All cellular lifeforms use two types of nucleic acids, RNA and DNA to store, propagate, and utilize their genetic information. RNA polymerases (RNAPs) synthesize RNA from ribonucleoside triphosphates (NTPs), whereas DNA polymerases (DNAPs) use 2′-deoxyribonucleoside triphosphates (2′dNTPs) to synthesize DNA. The RNA building blocks precede the DNA building blocks biosynthetically and possibly also evolutionarily^1,2^. Messenger RNA molecules function as information carriers in a single-stranded form, whereas ribosomal, transfer and regulatory RNAs adopt complex three-dimensional structures composed of double-stranded segments. The double-stranded RNAs favor A-form geometry where the ribose moiety of each nucleotide adopts the 3′-endo conformation (Fig. 1a). In contrast, DNA functions as a B-form double helix, where the deoxyribose of each nucleotide adopts the 2′-endo conformation (Fig. 1a, b). Hybrid duplexes between the RNA and DNA transiently form during transcription and adopt an A-form geometry because conformational preferences of the RNA strand outweigh those of a more flexible DNA strand. The sugar moieties of NTPs and 2′dNTPs equilibrate freely between the 3′- and 2′-endo conformations in solution with the overall bias typically shifted toward the 2′-endo conformers^3^. However, both NTPs and 2′dNTPs typically adopt the 3′-endo conformation in the active sites of the nucleic acid polymerases^4^.

RNAPs and DNAPs need to discriminate efficiently against the substrates with the non-cognate sugar. The intracellular levels of NTPs are in the range of hundreds of micromoles to several millimoles per liter and exceed those of the corresponding 2′dNTPs more than 10-fold^5–7^. Most DNAPs use bulky side-chain residues in their active sites to exclude the 2′OH of NTPs (reviewed in ref. ^8^). The steric gate residue, typically Gln/Glu in A-family DNAPs and Tyr/Phe in Y- and B-family DNAPs, stretches along the α-face of the deoxyribose moiety of an incoming 2′dNTP and forms a hydrogen bond between the backbone amide group and the 3′-OH group of 2′dNTP (Fig. 1c).

Selection against 2′dNTPs by RNAPs is a daunting challenge because 2′dNTPs are substructures of the corresponding NTPs. Single-subunit RNAPs (e.g., mitochondrial and bacteriophage T7 and N4 enzymes) are homologous and structurally similar to DNAPs. However, single-subunit RNAPs lack a steric gate and use a conserved Tyr residue to discriminate against 2′dNTPs^9,10^. Tyr forms a hydrogen bond with the 2′OH group of the NTP ribose (Fig. 1c)^11,12^ but mediates the selectivity by inhibiting the binding and incorporation of 2′dNTPs^9,13^. It is hypothesized that the formation of the Tyr–2′OH hydrogen bond upon the binding of NTPs counteracts an inhibitory interaction of the Tyr with another residue or a water molecule^10^. Noteworthy, a homologous Tyr hydrogen bonds with the steric gate Gln/Glu residue in A-family DNAPs (Fig. 1c)^14,15^.

The mechanism of discrimination against 2′dNTPs by the multi-subunit RNAPs (bacterial, archaeal, and eukaryotic nuclear RNAPs) is poorly understood. The combined structural evidence (reviewed in ref. ^16^) suggests that the 2′OH group can make polar contacts with three universally conserved amino acid side chains: β′Arg425, β′Asn458, and β′Gln929 (numbering of the *Escherichia coli* RNAP). β′Arg425 and β′Asn458 belong to the active site cavity and can interact with the 2′OH of NTPs in the open and closed active site (see below), whereas β′Gln929 is contributed by a mobile domain called the trigger loop (TL) and can only transiently interact with the 2′- and 3′-OH of NTPs in the semi-closed active site^17–19^ (Fig. 1d). Closure of the active site by the folding of two alpha-helical turns of the TL positions the triphosphate moiety of the substrate NTP inline for an attack by the 3′OH group of the RNA and accelerates catalysis ~10^4^ fold^20–23^. However, most structures of RNAP complexes with NTP analogs feature a semi-closed active site where the TL is helical up to β′Met932 (refs. ^17–19^) (Supplementary Table 1). Partial folding of the TL establishes contacts between β′Gln929 and the ribose moiety and stacking of β′Met932 with the nucleobase (Fig. 1d) but is insufficient to promote catalysis (3′OH → αP distance 5.4 Å^18^).

The relative contribution of the TL (β′Gln929 and β′Met932) and the active site cavity (β′Arg425 and β′Asn458) to the discrimination against 2′dNTPs remains hitherto uncertain. The closure of the active site makes only a 5- to 10-fold contribution to an overall 500- to 5000-fold selectivity in RNAPs from *E. coli*^23^ and *Saccharomyces cerevisiae*^24^. Consistently, the open active site of the *E. coli* RNAP retained a ~100-fold overall selectivity against 2′dNTPs^23^. However, the open active site of the *Thermus aquaticus* RNAP has been reported to be largely unselective^22^, and individual substitutions of the β′Asn458 with Ser in *E. coli* and *S. cerevisiae* reduced selectivity less than 5-fold^20,25^. Most importantly, although the universally conserved β′Arg425 closely approaches 2′OH of the NTP in several X-ray crystal structures^17–20,26^ (Supplementary Table 1) and has been highlighted as the sole residue mediating the selectivity against 2′dNTP in a computational study by Roßbach and Ochsenfeld^27^, its role has not been experimentally assessed.

In this study, we systematically investigated the effects of individual substitutions of the active site residues on the discrimination against 2′dNTPs in single nucleotide addition (SNA) assays and during processive transcript elongation by the *E. coli* RNAP. This analysis demonstrated that β′Arg425 is the major determinant of the selectivity against 2′dNTPs. We further analyzed the binding of 2′-deoxy substrates by in silico docking and X-ray crystallography of *Thermus thermophilus* RNAP. Our data suggest that the conserved Arg actively selects against 2′dNTPs by favoring their templated binding in the 2′-endo conformation that is poorly suitable for incorporation into RNA.

## Results

### β′Arg425 is the major determinant of the selectivity against 2′dGTP by *E. coli* RNAP

To investigate the mechanism of the discrimination against the 2′-deoxy substrates, we performed time-resolved measurements of the single nucleotide incorporation by the wild-type (WT) and variant *E. coli* RNAPs. Among several single substitutions of the key residues that contact NTP ribose (Fig. 1d), we selected four variant RNAPs that retained at least half of the WT activity at saturating concentration of NTPs. This approach minimized the possibility that the amino acid substitutions induced global rearrangements of the active site thereby complicating the interpretations of their effects on the sugar selectivity.

**Fig. 2:**
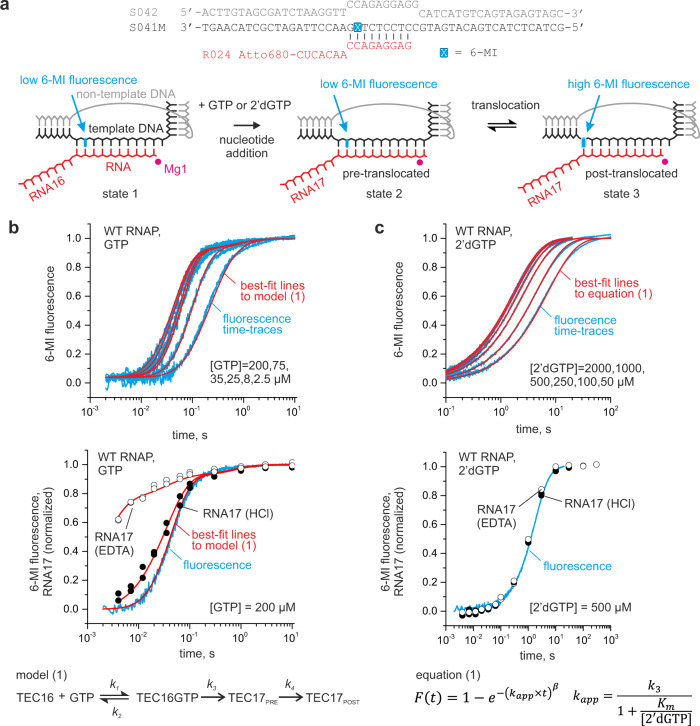
Time-resolved measurements of GTP and 2′dGTP utilization by the WT *E. coli* RNAP. **a** The nucleic acid scaffold employed in translocation and nucleotide addition assays. The fluorescence of a guanine analog 6-MI (cyan) was quenched by neighboring base pairs in the initial TEC (state 1) and the pre-translocated TEC that formed following the nucleotide incorporation (state 2) but increased when the 6-MI relocated to the edge of the RNA:DNA hybrid upon translocation (state 3). The template DNA, non-template DNA, RNA, and the catalytic Mg^2+^ ions are colored black, gray, red, and magenta, respectively. **b** GTP concentration series. The data were fit to model (1). **c** 2′dGTP concentration series. The data were fit to equation (1). The best-fit lines and fluorescence time-traces are colored red and cyan, respectively. The HCl and EDTA quenched data points are shown as closed and opened circles, respectively. All experiments were performed in duplicate with similar results, duplicate data were combined for the analysis. Source data are provided as a Source Data file.

Transcription elongation complexes (TECs) were assembled on synthetic nucleic acid scaffolds and they contained the fully complementary transcription bubble flanked by 20-nucleotide DNA duplexes upstream and downstream (Fig. 2a). The annealing region of a 16-nucleotide RNA primer was initially 9 nucleotides, permitting the TEC extended by one nucleotide to adopt the post- and pre-translocated states, but disfavoring backtracking^28^. The RNA primer was 5′ labeled with the infrared fluorophore ATTO680 to monitor the RNA extension by denaturing PAGE. The template DNA strand contained the fluorescent base analog 6-methyl-isoxanthopterin (6-MI) eight nucleotides upstream from the RNA 3′ end to monitor RNAP translocation along the DNA following nucleotide incorporation^29^.

**Fig. 3:**
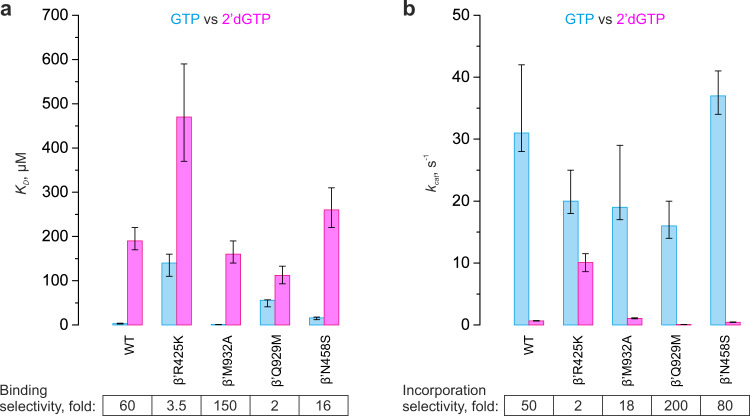
Nucleo-sugar selectivities of the WT and variant *E. coli* RNAP. **a** Equilibrium dissociation constants for the reversible binding of GTP and 2′dGTP. **b** Turnover numbers for incorporation of GMP and 2′dGMP. Cyan (GTP) and magenta (2′dGTP) bars indicate the best-fit values of the parameters estimated by the global fit of the data in Fig. 2b, c and Supplementary Figs. 1, 2 to model (1) (GTP) or equation (1) (2′dGTP). Duplicate data were combined for the analysis. Upper and lower bounds of the parameters values (error bars) were calculated by FitSpace routine of Kintek Explorer software (GTP) or Origin 2015 software (2′dGTP) at 10% increase in Chi^2^. Error bars define ranges of the parameters values that support a good fit of the model to data and are not measures of the biological variability. Numerical values of the parameters are indicated in Tables 1 and 2 and Supplementary Table 2.

We first measured GTP and 2′dGTP concentration series of the WT and altered RNAPs using a time-resolved fluorescence assay performed in a stopped-flow instrument (Fig. 2b, c). We used the translocation assay because it allowed rapid acquisition of concentration series, whereas measurements of concentration series by monitoring RNA extension in the rapid chemical quench-flow setup would be considerably more laborious. The concentration series data allowed the estimation of *k*_cat_ and the *Km* (Michaelis constant) for GTP and 2′dGTP. We then supplemented the concentration series with time-courses of GMP and 2′dGMP incorporation obtained using a rapid chemical quench-flow technique with EDTA as a quencher. EDTA inactivates the free GTP and 2′dGTP by chelating Mg^2+^ but allows a fraction of the already bound substrate to complete incorporation into RNA^30,31^. The EDTA quench experiment is thus equivalent to a pulse-chase setup and provides information about the rate of substrate dissociation from the active site of RNAP. A global analysis of the concentration series and EDTA quench experiments (i) allowed the estimation of the *K*_D_ for GTP dissociation from the active site and (ii) suggested that the *K*_D_ for the dissociation of 2′dGTP from the active site approximately equals the *Km* for 2′dGMP incorporation (see Supplementary Note). We further used inferred values of *k*_cat_ and *K*_D_ to compare the capabilities of the variant RNAPs to discriminate against 2′dGTP (Fig. 3).

WT RNAP displayed ~60-fold higher affinity for GTP than for 2′dGTP (Fig. 3a and Table 1). The β′R425K and β′Q929M substitutions decreased the selectivity at the binding step 17- and 30-fold, respectively, largely by decreasing the affinity for GTP. In contrast, β′N458S decreased the selectivity only 4-fold, whereas β′M932A increased the selectivity 2.5-fold. At saturating substrate concentrations, the WT RNAP incorporated GMP ~50-fold faster than 2′dGMP (Fig. 3b and Table 1). The β′R425K substitution decreased the selectivity 25-fold, primarily by accelerating the 2′dGMP incorporation. In comparison, the effects of other substitutions at the incorporation step were relatively small (Fig. 3b, Table 2, and Supplementary Table 2). β′M932A decreased the selectivity 3-fold, whereas β′N458S and β′Q929M increased the selectivity 1.5- and 4-fold, respectively. Noteworthy, β′Q929M decreased the rate of 2′dGMP incorporation 10-fold.

**Table 1:** Kinetic parameters for the substrate utilization by *E. coli* RNAPs (fit to model 1).

RNAP	Substrate	*k*_on_, µM^−1^ s^−1^	*k*_off_, s^−1^	*k*_cat_, s^−1^ (fast)	*k*_cat_, s^−1^ (slow)	Fast fraction, %	*K*_D_, µM
WT	GTP	2.5 (2.2–3.0)	7.7 (4.0–9.6)	31 (28–42)	2.6 (0.8–7.6)	88 (77–93)	3.1 (2.3–4.0)
WT	2′dGTP	>0.24	>45	1.4 (1.1–2.3)	0.35 (0.22–0.49)	52 (30–70)	190 (160–220)
β′R425K	GTP	0.5 (0.4–0.8)	72 (57–120)	20 (18–25)	1.3 (0.4–4.1)	86 (76–92)	140 (110–160)
β′R425K	2′dGTP	>0.10	>30	13 (11–18)	2.2 (0.7–5.4)	78 (50–90)	420 (330–480)
WT	3′dGTP	2.7 (2.2–4.4)	87 (70–140)	12 (11–13)	0.74 (0.5–1.4)	80 (75–85)	32 (28–38)

Reaction products were modeled as sums of independent contributions by fast and slow fractions of RNAP; contributions of each fraction were modeled as model (1). Upper and lower bounds were calculated at a 10% increase in Chi^2^ by the FitSpace routine of KinTek Explorer software.

**Table 2:** Kinetic parameters for the 2′dGTP utilization by *E. coli* RNAPs (fit to equation 1).

RNAP	*k*, s^−1^	*β* (stretching parameter)	*Km*, µM	Median reaction time, s	Median reaction rate, s^−1^
WT	0.62 (0.58–0.65)	0.83 (0.80–0.85)	190 (170–220)	1.04 (0.98–1.11)	0.67 (0.62–0.71)
β′R425K	9.7 (8.4–11.3)	0.90 (0.84–0.96)	470 (370–590)	0.069 (0.059–0.079)	10.1 (8.6–11.5)
β′M932A	1.01 (0.94–1.09)	0.85 (0.81–0.89)	160 (140–190)	0.64 (0.59–0.69)	1.07 (0.99–1.15)
β′Q929M	0.071 (0.066–0.076)	0.76 (0.73–0.79)	112 (93–133)	8.7 (8.1–9.3)	0.080 (0.074–0.085)
β′N458S	0.42 (0.39–0.45)	0.83 (0.80–0.87)	260 (220–310)	1.5 (1.4–1.7)	0.45 (0.41–0.49)

Upper and lower bounds were calculated at a 10% increase in Chi^2^ by Origin 2015 software.

Overall, these experiments suggested that the β′Arg425 residue plays a central role in the discrimination against 2′-deoxy substrates: β′Arg425 selectively facilitated binding of GTP and selectively inhibited the incorporation of 2′dGMP. In contrast, the role of β′Gln929 was complex: while β′Gln929 selectively facilitated the binding of GTP, it also selectively facilitated the incorporation of 2′dGMP.

### β′Arg425 inhibits the utilization of 2′dNTPs during processive transcript elongation

The time-resolved SNA assays described above are superior to any other currently available techniques for the quantitative assessment of the binding and incorporation of different substrates and the effects of active site residues therein. However, these assays have several limitations: the nucleotide incorporation was measured for static complexes stabilized in the post-translocated state by the artificially limited RNA:DNA complementarity and the effects are assessed only at a single, easy to transcribe, sequence position. To test if the conclusions drawn from the SNA assay remain valid during processive transcript elongation, we developed a semi-quantitative assay as follows.

**Fig. 4:**
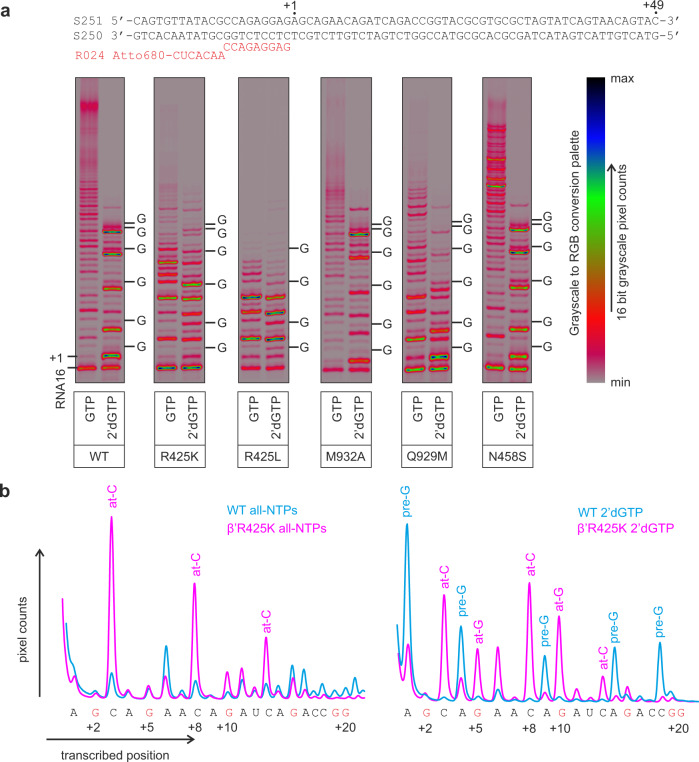
Utilization of 2′dGTP during processive transcription by the WT and variant *E. coli* RNAPs. **a** TECs were assembled using the scaffold shown above the gel panels and chased with 50 µM ATP, CTP, UTP, and GTP or 2′dGTP for 2 min at 25 °C. The positions of GMPs in resolved stretches of the transcribed sequence are marked along the right edge of gel panels; 16-bit grayscale scans were normalized using max pixel counts within each gel panel and pseudo-colored using RGB palette. **b** Lane profiles of transcription in all-NTPs and 2′dGTP chases by the WT (cyan) and β′R425K (magenta) RNAPs quantified from gels in **a**. Traces were manually aligned along the *X*-axis and scaled along the *Y*-axis using several sequence positions as references. All experiments were repeated in triplicate with similar results. Source data are provided as a Source Data file.

TECs were assembled on a nucleic acid scaffold with a 49-bp-long downstream DNA and chased with NTP mixtures containing 50 µM ATP, CTP, UTP, and GTP or 2′dGTP for 2 min at 25 °C. Transcription with 2′dGTP by the WT RNAP resulted in characteristic pauses at each sequence position preceding the incorporation of 2′dGMP (Fig. 4, pre-G sites). We used the amplitude of these accumulations as a semi-quantitative measure of the ability of RNAP to utilize 2′dGTP. Noteworthy, the interpretation of the processive transcription by some variant RNAPs was complicated by enhanced pausing after the incorporation of cytosine (Fig. 4b, at-C sites) and 2′dGMP (Fig. 4b, at-G sites) in certain sequence contexts. However, these additional pauses were unrelated to the utilization of 2′dGTP as a substrate and could be disregarded when comparing pre-G pauses that occurred upstream of all at-C and at-G pauses.

In contrast to the WT RNAP, the β′R425K enzyme did not pause prior to the incorporation of the 2′dGMP (Fig. 4), consistently with the significantly higher 2′dGMP incorporation rate observed in SNA assays (Fig. 3b). Moreover, the β′R425L RNAP also did not accumulate at the pre-G sites despite being strongly defective during processive transcription (Fig. 4a and Supplementary Fig. 3). These data suggest that the loss of selectivity is attributable to the absence of β′R425 rather than the presence of the Lys residue at the corresponding position.

The β′M932A RNAP paused noticeably less whereas the β′Q929M RNAP paused noticeably more than the WT RNAP at the pre-G sites (Fig. 4a and Supplementary Fig. 3) consistently with the 2-fold higher (β′M932A) and 10-fold lower (β′Q932M) *k*_cat_ for the 2′dGMP incorporation in the SNA experiments (Fig. 3b and Table 2). In contrast, the β′N458S RNAP was largely indistinguishable from the WT RNAP in its ability to utilize 2′dGTP in the processive transcription assay (Fig. 4 and Supplementary Fig. 3), presumably because this assay is not sensitive enough to resolve the ~1.5-fold difference in *k*_cat_ for the 2′dGMP incorporation (Fig. 3b and Table 2). Overall, the analysis of the 2′dGTP utilization during processive transcription of diverse sequences by the WT and variant RNAPs recapitulated the major effects observed in the SNA experiments.

Next, we tested the effects of the β′R425K, β′M932A, β′Q932M, and β′N458S substitutions on utilization of 2′dATP, 2′dCTP, and 2′dUTP (Supplementary Figs. 4–7). For each 2′dNTP, we designed a template where the 2′dNTP is incorporated several times early in transcription, thereby allowing unambiguous interpretation of the accumulation of RNAPs at sites preceding the 2′dNMP incorporation. An analysis of the utilization of 2′dATP, 2′dCTP, and 2′dUTP largely recapitulated the effects observed for 2′dGTP, except that β′N458S was markedly inferior to the WT RNAP in utilizing 2′dATP and 2′dUTP. Overall, these data demonstrated that the enhanced or diminished capabilities of the variant RNAPs to utilize 2′dGTP in the SNA assays reflected, in qualitative terms, their capabilities to utilize all four 2′dNTPs.

### β′Arg425 favors the binding of 2′dCTP in the 2′-endo conformation

The role of β′Arg425 in selectively promoting the binding of NTPs was easy to explain because β′Arg425 interacts with the 2′OH of the NTP analogs in several RNAP structures (Supplementary Table 1 and Fig. 1d). In contrast, the observation that β′Arg425 selectively inhibited the incorporation of 2′dNTPs could not be readily explained: our results show that the β′Arg425 substitutions promote the incorporation of the substrate that lacks the 2′OH group, which β′Arg425 would interact with. We hypothesized that, in the absence of the 2′OH, β′Arg425 interacted with something else and that this interaction slowed down the incorporation of 2′dNMPs into the nascent RNA. We further reasoned that the 3′OH group of the 2′dNTP was the most likely interacting partner of β′Arg425, an inference supported by MD simulations of *S. cerevisiae* RNAP II (ref. ^27^). However, the 3′OH group is positioned too far from β′Arg425 when the sugar moiety is in the 3′-endo conformation (Supplementary Table 1). We further hypothesized that the 3′OH could move to within the hydrogen bond distance of β′Arg425 if the deoxyribose moiety adopted a 2′-endo conformation.

**Fig. 5:**
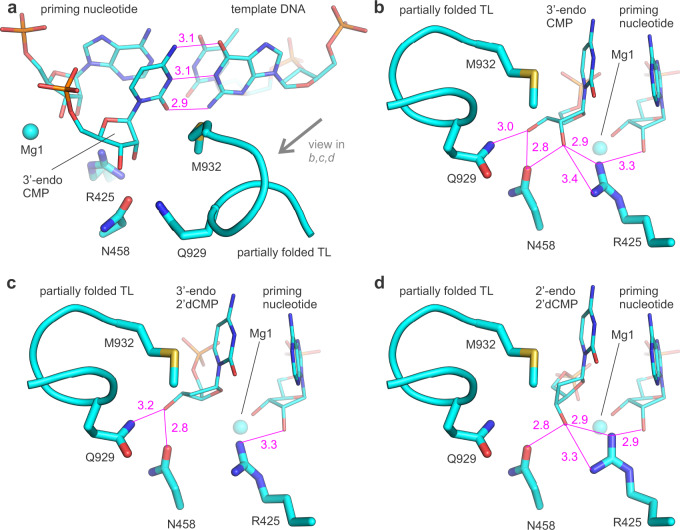
Docking models of *T. thermophilus* RNAP with ribo- and deoxyribonucleotides. In silico docking of the 3′-endo CMP (**a**, **b**), 3′-endo 2′dCMP (**c**), and 2′-endo 2′dCMP (**d**) in the semi-closed active site of *T. thermophilus* RNAP. Magenta numbers are interatomic distances in Å. Source data are provided as a Source Data file.

To test this hypothesis in silico, we removed cytidine-5′-[(α,β)-methyleno]triphosphate (CMPCPP) from the structural model of the initiation complex of *T. thermophilus* RNAP (PDB ID 4Q4Z)^18^ and docked 2′-endo 2′dCMP, 3′-endo 2′dCMP, and 3′-endo CMP (as a control) to the vacated active site (Fig. 5). We used nucleoside monophosphates instead of triphosphates as ligands because the docking software failed to correctly model interactions of triphosphate moieties with active site metal ions thereby complicating the interpretation of docking results (see “Methods”). The docking algorithm recovered high-scoring templated poses for CMP in 10 out of 10 runs, lower-scoring templated poses for 3′-endo 2′dCMP in 8 out of 10 runs and 2′-endo 2′dCMP in 5 out of 10 runs (Supplementary Table 3). The β′Arg425 side chain was kept flexible in the latter case because our manual assessment suggested that a sub-angstrom repositioning of β′Arg425 would be needed to accommodate the 2′-endo deoxyribose. We then fixed the β′Arg425 conformation observed in the highest-scoring templated pose and performed 10 additional docking runs. This time templated poses were recovered in 10 out of 10 runs. The robust recovery of templated poses suggested that the RNAP active site is well-suited for binding of the 2′-endo conformer of the 2′dCMP moiety via hydrogen bonding between the 3′OH and β′Arg425 (Fig. 5d), lending credence to our hypothesis.

**Fig. 6:**
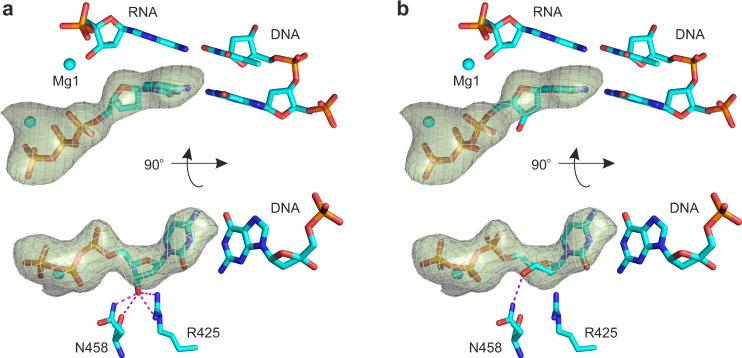
The 2′- and 3′-endo 2′dCTPs fitted into the omit map (3σ) of the RNAP-2′dCTP complex. **a** The 3′OH group of the 2′-endo conformer is positioned to form up to three hydrogen bonds and a polar interaction with RNAP atoms (magenta lines, interatomic distances 2.5–3.1 Å). **b** The 3′OH of the 3′-endo conformer can hydrogen bond with one RNAP atom. The 2′- and 3′-endo conformers were derived from PDB IDs 2HVW (resolution: 1.67 Å) and 4DQI (resolution: 1.69 Å), respectively, and fitted into the omit map by rotating bonds but preserving bond lengths and angles. Source data are provided as a Source Data file.

**Fig. 7:**
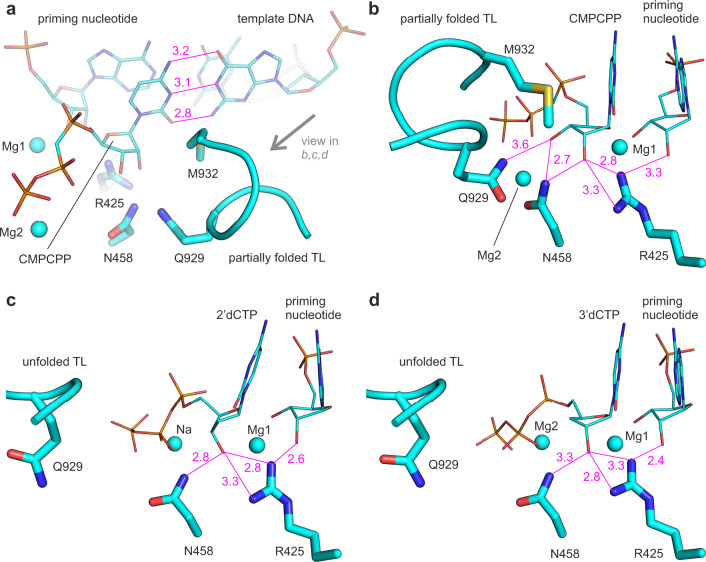
X-ray crystal structures of *T. thermophilus* RNAP with ribo- and deoxyribonucloside substrates. Crystallographically observed binding poses of CMPCPP (**a**, **b**), 2′dCTP (**c**), and 3′dCTP (**d**) in the active site of *T. thermophilus* RNAP. Magenta numbers are interatomic distances in Å. Panels **a** and **b** were prepared using PDB ID 4Q4Z^18^. Pre-catalytic complexes in **c** and **d** were trapped due to the low reactivity of deoxyribonucleoside substrates and the slow catalysis by RNAP in crystallo^52^. Source data are provided as a Source Data file.

To further test our hypothesis in crystallo, we solved the X-ray crystal structure of the initially transcribing complexes containing *T. thermophilus* RNAP, DNA, and 3-nt RNA primer with incoming 2′dCTP bound at the active site at 3.14 Å resolution. The structure displayed a well-resolved electron density of 2′dCTP and β′Arg425 closely approaching the deoxyribose moiety (Figs. 6, 7, Table 3, and Supplementary Fig. 8a). 2′dCTP was observed in the pre-insertion conformation that was unsuitable for catalysis because the α-phosphate was located 5.7 Å from the 3′OH of the RNA primer. The electron density was consistent with the interaction between the β′Arg425 residue and the 3′OH group of the deoxyribose in the 2′-endo conformation. While the resolution of the structure was insufficient to unambiguously determine the conformation of the deoxyribose, the 3′OH of the 2′endo conformer was positioned to hydrogen bond with up to three RNAP atoms (Fig. 6a), whereas the 3′OH of the 3′-endo conformer fitted into the omit map could only form a single hydrogen bond (Fig. 6b). Considering that 2′dNTPs intrinsically prefer the 2′-endo conformation in solution and when bound to RNA template in a non-enzymatic system^3^, these observations suggest that 2′dCTP predominantly adopts the 2′-endo conformation upon binding to the open active site of RNAP (TL unfolded, see below). Indeed, the 2′-endo 2′dCTP is intrinsically more favorable than the 3′-endo conformer and can form more favorable interactions with the amino acid residues in the open active site. Interestingly, the density for the metal ion complexed by the β- and γ-phosphates of 2′dCTP was weak and the coordination distances were longer than typically observed for Mg^2+^ in the corresponding position. We modeled this metal ion as Na^+^ rather than Mg^2+^ similarly to what has been proposed for DNA polymerase β^32^.

**Table 3:** Data collection and refinement statistics.

Complex	2′dCTP	3′dCTP
PDB code	6WOX	6WOY
*Data collection*		
Space group	C2	C2
Cell dimensions		
* a* (Å)	185.44	185.98
* b* (Å)	102.65	102.47
* c* (Å)	295.38	296.04
* β* (°)	98.91	98.87
Resolution (Å)	30–3.14	30–3.00
Total reflections	275,994	361,353
Unique reflections	89,931	106,804
Redundancy	3.1 (2.5)	3.4 (2.9)
Completeness (%)	94.3 (68.6)	96.9 (73.6)
*I* / *σ*	10.06 (1.44)	14.78 (1.73)
*R*_merge_	0.129 (0.521)	0.086 (0.553)
CC^1/2^	0.985 (0.829)	0.991 (0.855)
*Refinement*		
Resolution (Å)	30–3.14	30–3.00
*R*_work_	0.196	0.206
*R*_free_	0.261	0.219
No. of atoms	28,569	28,569
R.m.s. deviations		
Bond length (Å)	0.010	0.009
Bond angles (°)	1.22	1.12
Clashscore	15.04	13.19
Ramachandran favored, %	85.68	86.95
Ramachandran outliers, %	0.37	0.37

Data sets were collected at MacCHESS F1 line, Ithaca, NY.

Highest resolution shells are shown in parentheses.

The TL was completely unfolded in the structure of the initially transcribing complex with 2′dCTP, in contrast to a partially helical conformation typically observed in the structures of RNAP complexes with non-hydrolysable NTP analogs (Supplementary Table 1)^17–20^. The destabilization of the TL folding could be due to the absence of interactions between the TL β′Gln929 and the 3′OH of 2′dCTP, that in turn is an expected consequence of the 2′dCTP binding in the 2′-endo conformation (Fig. 5d). To test if the unavailability of the 3′OH group was indeed responsible for the destabilization of the TL folding, we solved the X-ray crystal structure of the initially transcribing complex of the *T. thermophilus* RNAP with 3′dCTP at 3.0 Å resolution. The structure displayed a well-resolved density of 3′dCTP and β′Arg425 closely approaching the 3′-deoxyribose moiety (Fig. 7d and Supplementary Fig. 8b). 3′dCTP was in the pre-insertion conformation unsuitable for catalysis because the α-phosphate was located 5.6 Å away from the 3′OH of the RNA primer. The overall pose of 3′dCTP was similar to that of CMPCPP: the 3′-deoxyribose likely adopted a 3′-endo conformation and the 2′OH group interacted with β′Arg425. However, the TL was completely unfolded, supporting our hypothesis that the unavailability of the 3′OH group was alone sufficient to significantly destabilize the folding of the first helical turn of the TL.

Overall, the comparative analysis of RNAP structures with CMPCPP, 2′dCTP, and 3′dCTP in combination with in silico docking experiments suggested that β′Arg425 inhibited the incorporation of 2′dNTPs by interacting with their 3′OH group and favoring the 2′-endo conformation of the deoxyribose moiety. At the same time, the structures did not provide a decisive answer as to why the 2′-endo conformations of 2′dNTPs were less suitable for incorporation into RNA than the 3′-endo conformations.

### The misplacement of the 3′OH only partially accounts for the inertness of 2′dNTPs

The X-ray structures and in silico modeling experiments suggested that interactions between the 3′OH of the deoxyribose moiety and the β′Arg425 or β′Gln929 residues were mutually exclusive. Accordingly, β′Arg425 could inhibit the incorporation of the 2′dNMP solely by slowing down the initial steps of the TL folding, by sequestering the 3′OH group and preventing its interaction with the TL β′Gln929. To test this hypothesis, we determined the incorporation rate of 3′dGMP by the WT RNAP (Supplementary Fig. 2c). We found that the *k*_cat_ for 3′dGMP incorporation was only 5-fold slower than the *k*_cat_ for GMP incorporation and 10-fold higher than the *k*_cat_ for 2′dGMP incorporation (Table 1). These data demonstrated that the sequestration of the 3′OH group accounted for no more than a 5-fold inhibition of the 2′dGMP incorporation by β′Arg425. The remaining 10-fold inhibition of the overall 50-fold inhibitory effect was contributed by some other features of the 2′-endo binding pose, as discussed below.

## Discussion

In this study, we performed a systematic analysis of the contributions of the active site residues of the multi-subunit RNAP to selecting NTPs over 2′dNTPs. We identified a conserved Arg residue, β′Arg425 (*E. coli* RNAP numbering) as the major determinant of the sugar selectivity. The β′Arg425 residue favored binding of GTP over 2′dGTP and selectively inhibited the incorporation of 2′dNMPs into RNA (Figs. 3, 4 and Table 1).

The enhancement of NTP binding by β′Arg425 is consistent with the observation that β′Arg425 is positioned to hydrogen bond with the 2′OH of the NTP substrate analogs in several RNAP structures (Supplementary Table 1) and with MD simulations of the *S. cerevisiae* RNAP II (ref. ^27^). However, the existing data fail to explain the inhibition of the 2′dNTP incorporation by β′Arg425. In search of an explanation, we performed in silico docking experiments and solved the X-ray crystal structures of transcribing *T. thermophilus* RNAP with the cognate 2′dCTP and 3′dCTP. These experiments revealed that β′Arg425 interacts with the 3′OH group of the 2′dNTP substrate and favors the 2′-endo conformation of the deoxyribose (Figs. 5d, 6a, and 7c). In contrast, the ribose of the cognate NTP substrate is stabilized in the 3′-endo conformation by multiple polar contacts and hydrogen bonds with β′Arg425, β′Asn458, and β′Gln929 (Figs. 1d, 5b, and 7b).

A comparison of the RNAP structures with bound CMPCPP, 2′dCTP, and 3′dCTP revealed very small changes in the β′Arg425 conformation (Supplementary Fig. 8c), arguing against a scenario where the repositioning of the β′Arg425 side chain upon the binding of 2′dNTPs slows the catalysis of the phosphodiester bond formation^27^. Accordingly, we reasoned that the preferential selection of the catalytically inert 2′-endo conformers of 2′dNTPs and the deformation of the catalytically labile 3′-endo conformers of 2′dNTPs by β′Arg425 were likely the major factors behind the slow incorporation of 2′dNMPs. However, it remained unclear why the 2′-endo conformers of the substrates were less suitable for the incorporation than the 3′-endo conformers.

We first explored the possibility that the sequestration of the 3′OH group by β′Arg425 makes it unavailable for the interaction with β′Gln929 of the TL (Figs. 5d and 7c), thereby destabilizing the TL-mediated closure of the active site. Indeed, the TL is partially folded in most structures with ribonucleotide substrate analogs (Fig. 5b, Supplementary Table 1)^17–20^, yet was completely unfolded in the structures we obtained with either 2′dCTP (Fig. 5c) or 3′dCTP (Fig. 5d). However, we found that the rate of the 3′dGMP incorporation by *E. coli* RNAP was 10-fold faster than the rate of the 2′dGMP incorporation (Table 1). Notably, the *T. thermophilus* RNAP also incorporates 3′dNMPs faster than 2′dNMPs^22^. These results suggested that the sequestration of the 3′OH group by β′Arg425 could account for no more than a 5-fold out of its 50-fold overall inhibitory effect.

Similarly, the effects of the β′Q929M substitution were inconsistent with the idea that the 3′OH capture by β′Arg425 could alone account for the slow rate of the 2′dNMP incorporation. If that were true, the β′Q929M variant should be relatively insensitive to the absence of the 2′OH group. However, the opposite was true: β′Q929M was only 2-fold slower in incorporating GMP than the WT RNAP, but 10-fold slower in incorporating 2′dGMP. We propose that β′Gln929 competes with β′Arg425 for the 3′OH group of the 2′dNTP substrate: β′Arg425 favors the catalytically inert 2′-endo conformer (Figs. 5d, 6a, and 7c), whereas β′Gln929 favors the catalytically labile 3′-endo conformer (Fig. 5c). As a result, β′Gln929 is more important during the incorporation of 2′dNMPs than NMPs.

Since the TL folding can account only for a fraction of the inhibitory effect, what other factors make the 2′-endo conformers of 2′dNTPs catalytically inert? It is noteworthy that the sugars of the attacking and substrate nucleotides adopt the 3′-endo conformation in all RNAPs and DNAPs during the nucleotide incorporation^4^. In other words, even the 3′ ends of DNA primers adopt the 3′-endo conformation to catalyze the incorporation of the 2′dNMPs into the DNA. Apparently, the A-form geometry is much better suited for the catalysis of the nucleotide condensation than the B-form geometry^3,33^. The better accessibility of the nucleophilic 3′OH group of the attacking nucleotide is likely the primary reason. The substrate then adopts the 3′-endo conformation to match the overall geometry of the A-form duplex and to avoid clashes with the attacking nucleotide^4^.

In general terms, the inertness of the 2′-endo conformation of 2′dNTPs can be partially attributed to the differences in the conformations of the triphosphate moieties that in turn originate from the differences in the bond angles at C4′ of the sugar between the 3′- and 2′-endo conformers (Fig. 1b). We term this inhibitory component as C4′-geometry-dependent effects. However, it is impossible to further refine this hypothesis at present because the superimposition of different structures suggests a considerable conformational diversity of triphosphate moieties in the RNAP active site (Fig. 4 in ref. ^16^). High-resolution and time-resolved structural studies of nucleotide incorporation by the multi-subunit RNAPs would be necessary to determine the reasons behind the inertness of the 2′-endo conformers of 2′dNTPs.

Noteworthy, the conserved Arg is one of only seven catalytic residues that are conserved in the superfamily of “two-β-barrel” RNAPs^34,35^ that includes the multi-subunit RNAPs and very distantly related cellular RNA-dependent RNAPs (RdRps) involved in the RNA interference (Supplementary Fig. 9). Accordingly, the common ancestor of the two-β-barrel RNAPs could conceivably discriminate against 2′dNTPs and therefore likely evolved in the presence of both NTPs and 2′dNTPs. This inference lends credence to the hypothesis that proteins evolved in primordial lifeforms that already possessed both RNA and DNA^36,37^.

Viral RdRps (members of the “right-hand” superfamily of nucleic acid polymerases) are not homologous to multi-subunit RNAPs but share some elements of their sugar selection strategies. It appears that the 3′OH of the substrate NTP facilitates the active site closure in both classes of enzymes. In multi-subunit RNAPs, 3′OH facilitates the TL folding via the interaction with β′Gln929 (ref. ^18^), whereas in viral RdRps, 3′OH initiates the closure by sterically clashing with Asp238 (poliovirus RdRp numbering)^38^. In both classes of enzymes, 2′dNTPs adopt a 2′-endo pose wherein the 3′OH is misplaced and cannot readily facilitate the closure of the active site, explaining low reactivities of 2′dNTPs. However, 3′dNTPs are better substrates than 2′dNTPs also for viral RdRps^30^ suggesting that the low reactivity of the 2′-endo 2′dNTPs additionally relies on C4′-geometry-dependent effects (see above), which lead to a suboptimal conformation of the triphosphate moiety, a suboptimal geometry of the transition state, or both.

Multi-subunit RNAPs and viral RdRps converged on using the 2′-endo binding pose to discriminate against 2′dNTPs. In doing so these enzymes accentuate the intrinsic preferences of 2′dNTPs to retain the inert 2′-endo conformation upon binding to the A-form template in the non-enzymatic system^3^. However, the exact implementations of the selection mechanisms are distinct. In multi-subunit RNAPs, the 2′-endo pose is stabilized by the 3′OH/β′Arg425 attraction, whereas in viral RdRps, the 2′-endo pose is imposed by the 3′OH/Asp238 steric clash^38^. Multi-subunit RNAPs employ the conformational selection and preferentially sample the catalytically labile 3′-endo conformers of NTP and the catalytically inert 2′-endo conformers of 2′dNTPs. In contrast, viral RdRps likely rely exclusively on the induced fit^30^ and bind the catalytically inert 2′-endo conformers of either NTPs or 2′dNTPs. Following the initial binding, only NTPs can efficiently isomerize into catalytically labile 3′-endo conformers, ultimately repositioning the Asp238 and switching the RdRp active site on^38^. These principal differences in the substrate selection mechanisms can be potentially exploited for the concept-based design of sugar-modified substrate analog inhibitors of viral RdRps such as Remdesivir, one of few drugs currently available for COVID-19 treatment^39,40^.

In summary, our data show that the universally conserved Arg residue plays a central role in selecting NTPs over 2′dNTPs by the multi-subunit RNAPs. When NTP binds in the RNAP active site, its ribose adopts the 3′-endo conformation that positions the 3′OH group to interact with the universally conserved Gln residue of the TL domain and promotes the closure of the active site, whereas the triphosphate moiety can undergo rapid isomerization into the insertion conformation leading to efficient catalysis. The interaction of the conserved Arg residue with the 2′OH of the NTPs selectively enhances their binding more than 100-fold and renders RNAP saturated with NTPs in the physiological concentration range. In contrast, the interaction of the conserved Arg with the 3′OH of the 2′dNTP substrates shapes their deoxyribose moiety into the catalytically inert 2′-endo conformation where the 3′OH cannot promote closure of the active site and substrate incorporation is additionally inhibited by the unfavorable geometry of the triphosphate moiety. The deformative action of the conserved Arg on the 2′dNTP substrates is an elegant example of active selection against a substrate that is a substructure of the correct substrate.

## Methods

### Reagents and oligonucleotides

DNA and RNA oligonucleotides were purchased from Eurofins Genomics GmbH (Ebersberg, Germany) and IBA Biotech (Göttingen, Germany). DNA oligonucleotides and RNA primers are listed in Supplementary Table 4. NTPs, 2′dATP, 3′dGTP, and CMPCPP were from Jena Bioscience (Jena, Germany); 2′dGTP, 2′dUTP, and 2′dCTP were from Bioline Reagents (London, UK). TECs extended with 3′dGMP did not extend further upon the addition of the next substrate NTP suggesting that 3′dGTP stocks were free of GTP. TECs extended with 2′dGMP, 2′dATP, and 2′dUTP migrated faster in the denaturing PAGE than TECs extended with the corresponding NMPs suggesting that 2′dGTP, 2′dATP, and 2′dUTP stocks were free of the corresponding NTPs. 2′dCTP stocks were slightly contaminated by CTP as evident from the WT and β′M932A RNAPs gels in Supplementary Fig. 6. These low *K*_D_ RNAPs scavenged and depleted the trace amounts of CTP when transcribing the first CMP encoding position but incorporate exclusively 2′dCMP when transcribing CMP encoding positions further downstream. While it was possible to deplete the contaminating CTP by pre-treatment with the unlabeled TEC, we opted to present the experiment with a slightly contaminated 2′dCTP as a showcase of our capabilities to detect contaminations of 2′dNTPs with NTPs.

### Proteins

*E. coli* RNAPs were expressed in the *E. coli* strain T7 Express lysY/Iq (New England Biolabs, Ipswich, MA, USA) and purified by Ni-, heparin, and Q-sepharose chromatography as described previously^41^. RNAPs were dialyzed against storage buffer (50% glycerol, 20 mM Tris-HCl pH 7.9, 150 mM NaCl, 0.1 mM EDTA, 0.1 mM DTT) and stored at −20 °C. Plasmids used for protein expression are listed in Supplementary Table 5. *T. thermophilus* RNAP holoenzyme was prepared as described previously^18^.

### TEC assembly

TECs were assembled by a procedure developed by Komissarova et al.^42^. An RNA primer was annealed to the template DNA, and incubated with 1.5 µM RNAP for 10 min at 25 °C in TB10 buffer (10 mM MgCl_2_, 40 mM HEPES-KOH pH 7.5, 80 mM KCl, 5% glycerol, 0.1 mM EDTA, and 0.1 mM DTT) and with 2 µM of the non-template DNA for 20 min at 25 °C. For TECs used in nucleotide addition measurements, RNA was the limiting component at 1 µM (final concentration), and the template strand was used at 1.4 µM, whereas for TECs used in the translocation assay the template strand was limiting at 1 µM, and RNA was added at 1.4 µM.

### In vitro transcription reactions, processive transcript elongation

The transcription reactions were initiated by the addition of 10 µl of the assembled TEC (0.45 µM) to 10 µl of the substrate mixture (100 µM of each NTP or 2′dNTP), both solutions were prepared in TB10 buffer. In total, five mixtures containing NTPs and 2′dNTPs in different combinations were employed. Four chase mixtures contained three NTPs and one 2′dNTP (2′dATP-, 2′dCTP-, 2d′GTP-, and 2′dUTP-chase) whereas the control chase mixture contained four NTPs. The final concentration of NTPs and 2′dNTPs in the reaction mixtures was 50 µM each. The reactions were incubated for 2 min at 25 °C and quenched with 40 µl of Gel Loading Buffer (94% formamide, 20 mM Li_4_-EDTA, and 0.2% Orange G). RNAs were separated on 16% denaturing polyacrylamide gels and visualized with an Odyssey Infrared Imager (Li-Cor Biosciences, Lincoln, NE, USA); band intensities were quantified using the ImageJ software^43^.

### Time-resolved nucleotide addition measurements

Time-resolved measurements of nucleotide addition were performed in an RQF 3 quench-flow instrument (KinTek Corporation, Austin, TX). The reaction was initiated by the rapid mixing of 14 µl of 0.2 µM TEC with 14 µl of GTP (400, 2000, or 4000 µM) or 2′dGTP (400, 2000, or 4000 µM) or 3′dGTP (5, 20, 50, 200, 1000 µM). Both TEC and substrate solutions were prepared in TB10 buffer. The reaction was allowed to proceed for 0.004–300 s at 25 °C and quenched by the addition of 86 µl of 0.45 M EDTA or 0.5 M HCl. EDTA quenched reactions were mixed with 171 µl of Gel Loading Buffer. HCl quenched reactions were immediately neutralized by adding 171 µl of Neutralizing-Loading Buffer (94% formamide, 290 mM Tris base, 13 mM Li_4_EDTA, 0.2% Orange G). RNAs were separated on 16% denaturing polyacrylamide gels and visualized with an Odyssey Infrared Imager (Li-Cor Biosciences, Lincoln, NE, USA); band intensities were quantified using the ImageJ software^43^.

### Time-resolved fluorescence measurements

Measurements were performed in an Applied Photophysics (Leatherhead, UK) SX.18MV stopped-flow instrument at 25 °C. The 6-MI fluorophore was excited at 340 nm and the emitted light was collected through a 400-nm longpass filter. The nucleotide addition reactions were initiated by mixing 60 µl of 0.2 µM TEC in TB10 buffer with 60 µl of GTP (5–4000 µM) or 2′dGTP (100–4000 µM) in TB10 buffer. At least three individual traces were averaged for each concentration of the substrate.

### Data analyses

Time-resolved GMP incorporation data (HCl and EDTA quenched reactions) and the translocation timetraces were simultaneously fitted to a three-step model (model 1) using the numerical integration capabilities of the KinTek Explorer software^44^ (KinTek Corporation, Austin, TX) largely as described previously^45^. The model postulated that the initial TEC16 reversibly binds the GTP substrate, undergoes the irreversible transition to TEC17 upon incorporation of the nucleotide into RNA, followed by the irreversible translocation. The EDTA quenched reactions were modeled using the pulse-chase routine of the Kin-Tek Explorer software. Time-resolved 2′dGMP incorporation concentration series (translocation timetraces) were globally fitted to a stretched exponential function (equation 1) using Origin 2015 software (OriginLab, Northampton, MA, USA): the exponent followed a hyperbolic dependence on the 2′dGTP concentration; *Km*, rate constant *k* and the stretching parameter *β* were shared by all curves in the dataset. A detailed description of the data analyses is presented in Supplementary Note.

### Docking experiments

An RNAP fragment comprising amino acid residues and the template DNA within 20 Å from the active-site-bound CMPCPP was extracted from the X-ray crystal structure of the initiation complex of *T. thermophilus* RNAP (PDB ID 4Q4Z)^18^. The substrate binding site was vacated by removing CMPCPP. 3D structures of the 3′-endo CTP, 3′-endo 2′dCMPNPP, and 2′-endo 2′dCMP were extracted from PDB ID 3BSO (1.74 Å)^46^, 4O3N (1.58 Å)^47^, and 3FL6 (1.17 Å)^48^, respectively. Phosphate moieties were rebuilt using Discovery Studio 4.5 (Accelrys, San Diego, CA, USA) to produce 3′-endo CTP, CMP, 2′dCTP, 2′dCMP, 2′-endo 2′dCTP, and 2′dCMP. Ligands and the RNAP fragment were prepared for docking using AutoDock tools^49^. AutoDock Vina 1.1.2 docking runs were performed in a 16 × 20 × 20 Å search space centered at 183, 6, 83 Å (coordinate space of PDB ID 4Q4Z) using the default scoring function^50^. The docking was performed using 12 simultaneous computational threads; 20 binding poses were recorded for each run. Binding poses involving Watson–Crick pairing between the substrate and the template DNA (templated poses) were manually selected and extracted for further analysis (Supplementary Table 3). Our initial docking trials revealed that docking of nucleoside monophosphates produced the most robust and quantitatively interpretable results. Thus, the docking algorithm failed to recover templated poses for nucleosides without phosphate groups. The docking algorithm also failed to position the triphosphate moiety to coordinate metal ion number two and instead attempted to maximize its contacts with the protein. As a result, the recovered conformations of the triphosphate moieties differed from those observed in crystal structures. Considering the high impact of the triphosphate moiety on the ligand binding score and our assessment that the triphosphate moiety was docked incorrectly, we opted to limit the systematic investigation of the interaction between RNAP and the sugar moieties of nucleosides to docking nucleoside monophosphates.

### Preparation of the promoter DNA scaffold for the crystallization

The non-template DNA strand (5′-TATAATGGGAGCTGTCACGGATGCAGG-3′) was annealed to the template DNA strand (5′-CCTGCATCCGTGAGTGCAGCCA-3′) in 40 μl of 10 mM Tris-HCl (pH 8.0), 50 mM NaCl, and 1 mM EDTA to the final concentration of 1 mM. The solution was heated at 95 °C for 10 min and then gradually cooled to 22 °C.

### Crystallization of the *T. thermophilus* RNAP initially transcribing complexes

The crystals of the RNAP and promoter DNA complex were prepared as described previously^51,52^. The RNAP and promoter DNA complex was prepared by mixing 24 µl of 18 µM *T. thermophilus* holoenzyme (in 20 mM Tris-HCl, pH 7.7, 100 mM NaCl, and 1% glycerol) and 0.65 µl of 1 mM DNA scaffold and incubated for 30 min at 22 °C. Crystals were obtained by using hanging drop vapor diffusion by mixing equal volume of RNAP–DNA complex solution and crystallization solution (100 mM Tris-HCl, pH 8.7, 200 mM KCl, 50 mM MgCl_2_, 10 mM Spermine tetra-HCl, and 10% PEG 4000) and incubating at 22 °C over the same crystallization solution. The crystals were cryoprotected by soaking in same constituents as the crystallization solution with stepwise increments of PEG4000 and (2R,3R)-(—)-2,3-butanediol (Sigma-Aldrich) to final concentrations of 25% and 15%, respectively. The crystals were sequentially transferred to the final cryoprotection solution containing 1 mM primer 5′-GpCpA-3′ for 1 h and then transferred to the cryoprotection solution containing either 4 mM 2′dCTP or 3′dCTP for 30 s to trap the pre-catalytic complexes. The crystals were harvested and flash frozen in liquid nitrogen.

### X-ray data collections and structure determinations

The X-ray datasets were collected at the Macromolecular Diffraction at the Cornell High Energy Synchrotron Source (MacCHESS) F1 beamline (Cornell University, Ithaca, NY, USA) and structures were determined as previously described^51,52^ using the following crystallographic software: HKL2000^53^, Phenix^54^, and Coot^55^. Structure figures were prepared using PyMOL (Schrödinger, LLC, New York, NY, USA).

### Reporting summary

Further information on research design is available in the Nature Research Reporting Summary linked to this article.

## Supplementary information

Supplementary Information

Peer Review File

Reporting Summary

## Data Availability

The data that support this study are available from the corresponding authors upon reasonable request. X-ray crystallographic structure coordinates and their structural factors have been deposited in the RCSB Protein Data Bank (https://www.rcsb.org/) with accession codes 6WOX and 6WOY. Source data are provided with this paper.

## Source data

Source Data
