# Supplementary material for: The mechanism of the nucleo-sugar selection by multi-subunit RNA polymerases
Source: Nat Commun. 2021 Feb 4;12:796. doi: 10.1038/s41467-021-21005-w (PMC7862312; doi:10.1038/s41467-021-21005-w)
Supplement: Supplementary file 1 — Supplementary Information [file 41467_2021_21005_MOESM1_ESM.pdf]

Supplementary Information

The mechanism of the nucleo-sugar selection by multi-subunit RNA polymerases

Janne J. Mäkinen[1], Yeonoh Shin[2], Eeva Vieras[1], Pasi Virta[3], Mikko Metsä-Ketelä[1], Katsuhiko S. Murakami[2]*, Georgiy A. Belogurov[1]*
[1]University of Turku, Department of Biochemistry, Turku, Finland
[2]Department of Biochemistry and Molecular Biology, The Center for RNA Molecular Biology, The Pennsylvania State University, University Park, PA 16802.
[3]Department of Chemistry, University of Turku, FIN-20014 Turku, Finland
* To whom correspondence should be addressed: kum14@psu.edu (K.S.M.) and gebelo@utu.fi (G.A.B).

Supplementary Table 1. Distances between β'Arg425 and proximal polar moieties.

PDB ID*	Nominal resolution Å	Closest distance to the β'Arg425 guanidinium group, Å						TL helical up to:	Description
		substrate			RNA 3'end		β'Y457 main chain O		
		2'OH	3'OH	O4'	2'OH	3'OH			
4Q4Z	2.9	2.8 (CMPCPP)	4.5	2.8	3.3	3.7	2.8	β'M932	*Tth*, IC
5X22	3.35	3.2 (CMPCPP)	4.6	3.1	3.0	4.6	3.5	β'M932	*Tth*, ITC
2NVT	3.36	3.1 (GMPCPP)	5.0	4.9	2.4	4.5	4.1	β'Q929	*Sce* RNAPII, TEC
4OIO	3.1	3.3 (CMPCPP)	4.3	3.1	2.8	4.6	3.0	unfolded	*Tth*, IC
5X21 **	3.32	3.3 (PUM)	4.4	3.5	2.6	4.5	3.8	β'M932	*Tth*, ITC, +PUM
4A3E	3.4	3.3 (AMPCPP)	5.3	3.3	3.2	5.6	3.2	β'M932	*Sce* RNAPII, ITC
2PPB	3.0	3.7 (AMPCPP)	4.7	3.4	2.5	4.3	6.4****	unfolded***	*Tth*, TEC, +Stl
2O5J **	3.0	3.8 (AMPCPP)	3.8	2.9	2.9	5.4	6.4****	β'I937	*Tth*, TEC
6HLR	3.18	4.0 (GMPCPP)	4.0	4.0	2.5	4.5	3.6	β'M932	RNAPI, TEC
6WOX	3.14	n.a. (2'dCTP)	2.8	3.8	2.6	4.6	2.7	unfolded	*Tth*, ITC
6WOY	3.0	2.8 (3'dCTP)	n.a.	3.1	2.4	3.8	3.0	unfolded	*Tth*, ITC
4Q5S	3.0	4.4 (3'CMP)	5.4	2.8	2.3	n.a.	2.9	β'M932	Tth, ITC, PRE
2O5I	2.5	n.a.	n.a.	n.a.	2.5	3.8	5.8****	unfolded	*Tth*, TEC
3S14	2.85	n.a.	n.a.	n.a.	2.8	5.4	2.9	unfolded	*Sce* RNAPII, ITC
6C6T	3.5	n.a.	n.a.	n.a.	2.4	4.0	3.4	unfolded	*Eco*, TEC
6GMH	3.1	n.a.	n.a.	n.a.	2.7	4.7	3.6	unfolded	*Hsa* RNAPII, TEC

* The survey was limited to RNAP complexes with nucleic acids with the nominal resolution ≤3.5 Å. **β'Arg425 sidechain density is weak *** Unfolded due to the presence of the streptolydigin (Stl). ****Main chain of the active site loops is modeled differently from other structures; 2PBB and 2O5J are 2O5I soaked with ligands. *Tth*, *Thermus thermophilus*; *Sce*, *Saccharomyces cerevisiae*; *Eco*, *Escherichia coli*; *Hsa*, *Homo sapiens*; IC, initiation complex, ITC, initially transcribing complex; TEC, transcript elongation complex; PRE, pre-translocated state; PUM, pseudouridimycin; CMPCPP, cytidine-5'-[(α,β)-methyleno]triphosphate; GMPCPP, guanosine-5'-[(α,β)-methyleno]triphosphate; AMPCPP, adenosine-5'-[(α,β)-methyleno]triphosphate.

Supplementary Table 2. Kinetic parameters for GTP utilization by *E. coli* RNAPs (fit to model 1).

RNAP	k_{on} , μ$M^{-1}s^{-1}$	k_{off} , s^{-1}	k_{cat} , s^{-1} (fast)	k_{cat} , s^{-1} (slow)	*fast fraction*, %	k_{tra} , s^{-1}	K_D , μM
WT	2.5 (2.2 – 3.0)	7.7 (4.0 – 9.6)	31 (28 – 42)	2.6 (0.8 – 7.6)	88 (77 – 93)	96 (61 – 140)	3.1 (2.3 - 4.0)
β'R425K	0.5 (0.4 – 0.8)	72 (57 – 120)	20 (18 – 25)	1.3 (0.4 – 4.1)	86 (76 – 92)	220 (110 – 1600)	140 (110 – 160)
β'M932A	2.8 (2.2 – 8.4)	3.1 (0.8 – 5.4)	19 (17 – 29)	3.6 (0.2 – 11)	86 (45 – 98)	96 (61 – 230)	1.1 (0.6 – 1.4)
β'Q929M	1.1 (0.9 – 1.6)	62 (49 – 92)	16 (14 – 20)	1.3 (0.6 – 2.3)	79 (73 – 87)	140 (90 – 430)	56 (41 – 57)
β'N458S	1.7 (1.5 – 2.0)	28 (22 – 35)	37 (34 – 41)	1.2 (0.1 – 5.5)	94 (89 – 96)	170 (121– 260)	16 (12 – 18)

The reaction products were modelled as sums of independent contributions by the fast and slow fractions of RNAP; contributions of each fraction were modeled as model (1). Upper and lower bounds of the parameters were calculated at a 10% increase in Chi^2 by the FitSpace routine of the KinTek Explorer software.

Supplementary Table 3. Recovery of templated poses for CMP and 2'dCMP in docking experiments.

Receptor	*T. thermophilus* RNAP initiation complex, PDB ID 4Q4Z, partially folded TL.			
Original ligand	3'-endo CMPCPP			
Docked ligand	3'-endo CMP PDB ID 3BSO	3'-endo 2'dCMP PDB ID 4O3N	2'-endo 2'dCMP PDB ID 3FL6	2'-endo 2'dCMP PDB ID 3FL6
Side chains	rigid	rigid	β'R425 flexible (*Tth* β'R704)	β'R425 altered (*Tth* β'R704)
Run	Recovered templated poses and their binding scores (kcal/mol, Autodock Vina scoring function)			
1	-7.8, -7.7	-6.9	-	-7.1
2	-7.8, -7.6	-	-	-7.0, -6.9
3	-7.9, -7.8	-6.7	-	-7.2, -7.0
4	-7.8, -7.7	-6.9, -6.5	-6.9	-7.0
5	-7.8, -7.6	-	-6.7	-7.0
6	-7.9, -7.7	-6.8, -6.7	-	-6.9
7	-7.6, -7.5	-6.3	-7.1, -7.1	-7.0
8	-7.6, -7.5	-7.1	-	-7.0
9	-7.9, -7.7	-7.0	-6.8	-6.9
10	-7.8, -7.6	-6.8	-6.6	-7.1
Pose recovery, CI95%*	75 – 100%	48 – 95%	24 – 76%	75 – 100%
Binding score, CI95%**	-7.87 to -7.71 kcal/mol	-7.01 to -6.61 kcal/mol	-7.06 to -6.58 kcal/mol	-7.09 to -6.95 kcal/mol

* Calculated using adjusted Wald method. Recovery was treated as binary data: multiple poses per run were counted as a single recovery event. **Counting only one highest scoring pose per run.

Supplementary Table 4. DNA oligonucleotides and RNA primers used in this study.

Name	type	Sequence (5'→3')	Employment in Figures
S041M*	tDNA	GCTACTCTACTGACATGATGCCTCCTCTXGAACCTTAGATCGCTACAAGT	2,3, S1,2
S042	ntDNA	ACTTGTAGCGATCTAAGGTTCCAGAGGAGGCATCATGTCAGTAGAGTAGC	2,3, S1,2
S250	tDNA	GTACTGTTACTGATACTAGCGCACGCGTACCGGTCTGATCTGTTCTGCTCTCCTCTGGCGTATAACACTG	4, S3
S251	ntNA	CAGTGTTATACGCCAGAGGAGAGCAGAACAGATCAGACCGGTACGCGTGCGCTAGTATCAGTAACAGTAC	4, S3
S341	tDNA	TGGTGATCTGGCTTATCTCTGGTGATAGTAGCTCCTCTTAAACCTTAGATCG	S4,5
S342	ntDNA	CGATCTAAGGTTTAAGAGGAGCTACTATCACCAGAGATAAGCCAGATCACCA	S4,5
S343	tDNA	TGGTGATCTGGCTTCTATCTGGTGATGTAGTCTCCTCTGCAACCTTAGATCG	S6,7
S344	ntDNA	CGATCTAAGGTTGCAGAGGAGACTACATCACCAGATAGAAGCCAGATCACCA	S6,7
R002	RNA	Atto680-CACUAACUAAGAGGAG	S4,5
R024	RNA	Atto680-CUCACAACCAGAGGAG	2-4, S1-3
R140	RNA	Atto680-CAACACAGCAGAGGAG	S6,7

*****X = 6-methyl-isoxanthopterin

Supplementary Table 5. *E. coli* protein expression vectors used in this study.

Name	Description	Source/reference
pVS10	wild-type RNAP (T7p-α-β-β'_His$_6$-ω)	Ref. [41]
pAM012	β'M932A RNAP (T7p-α-β-β'[M932A]_TEV_His$_{10}$-ω)	Ref. [29]
pAM017	β'R425L RNAP (T7p-α-β-β'[R425L]_TEV_His$_{10}$-ω)	this work
pAM018	β'R425K RNAP (T7p-α-β-β'[R425K]_TEV_His$_{10}$-ω)	this work
pJM017	β'Q929M RNAP (T7p-α-β-β'[Q929M]_TEV_His$_{10}$-ω)	this work
pIA528+pIA839	β'N458S RNAP (T7p-α-β-β'[N458S]_His$_6$) + *araB*p-ω	Ref. [25]

Sequences of the plasmids are included in a Source Data file.

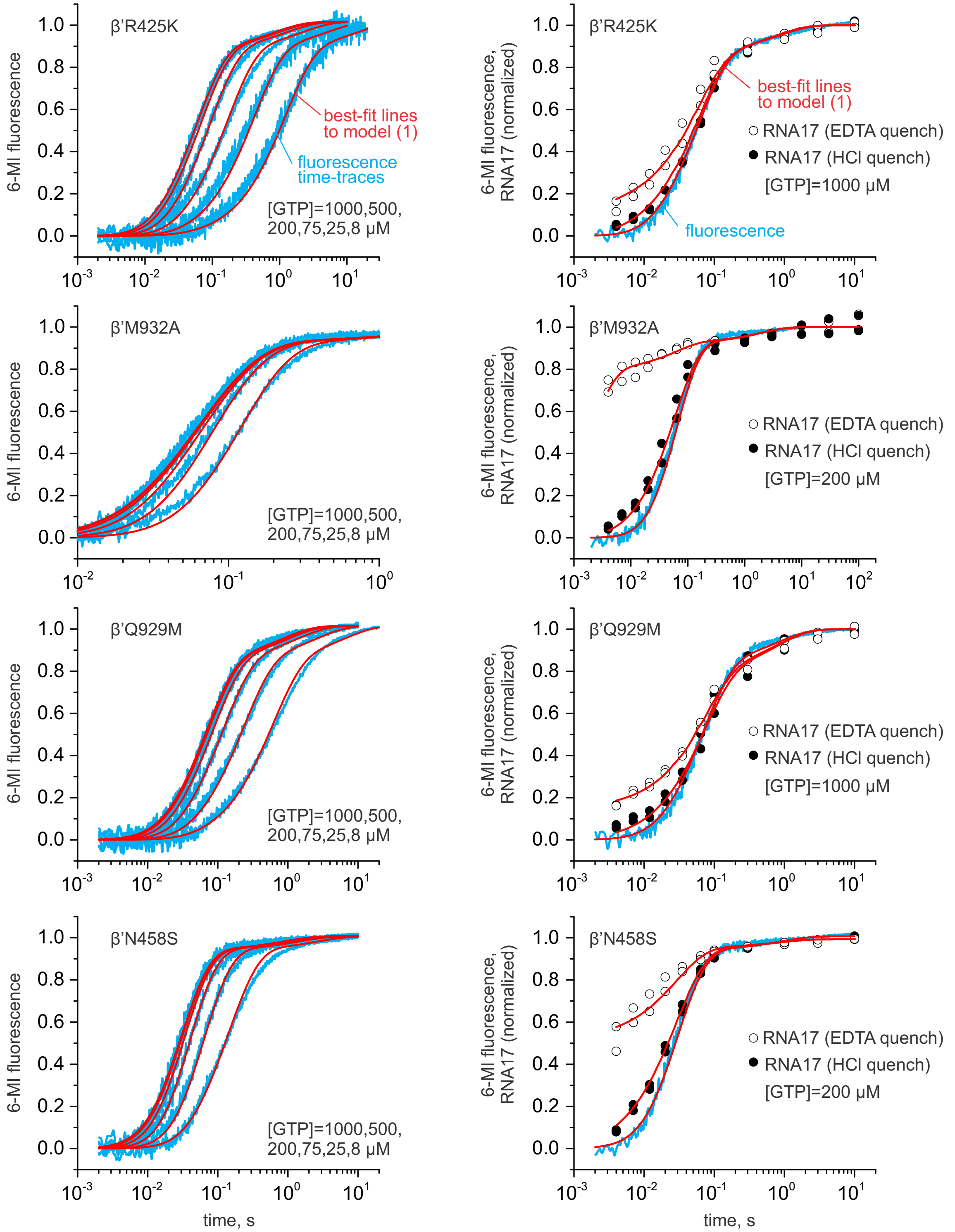

Supplementary Figure 1: GTP concentration series by variant *E. coli* RNAPs. The best-fit lines and fluorescence time-traces are colored red and cyan, respectively. The HCl and EDTA quenched data points are shown as closed and opened circles, respectively. All experiments were performed in duplicate with similar results, duplicate data were combined for the analysis. Source data are provided as a Source Data file.

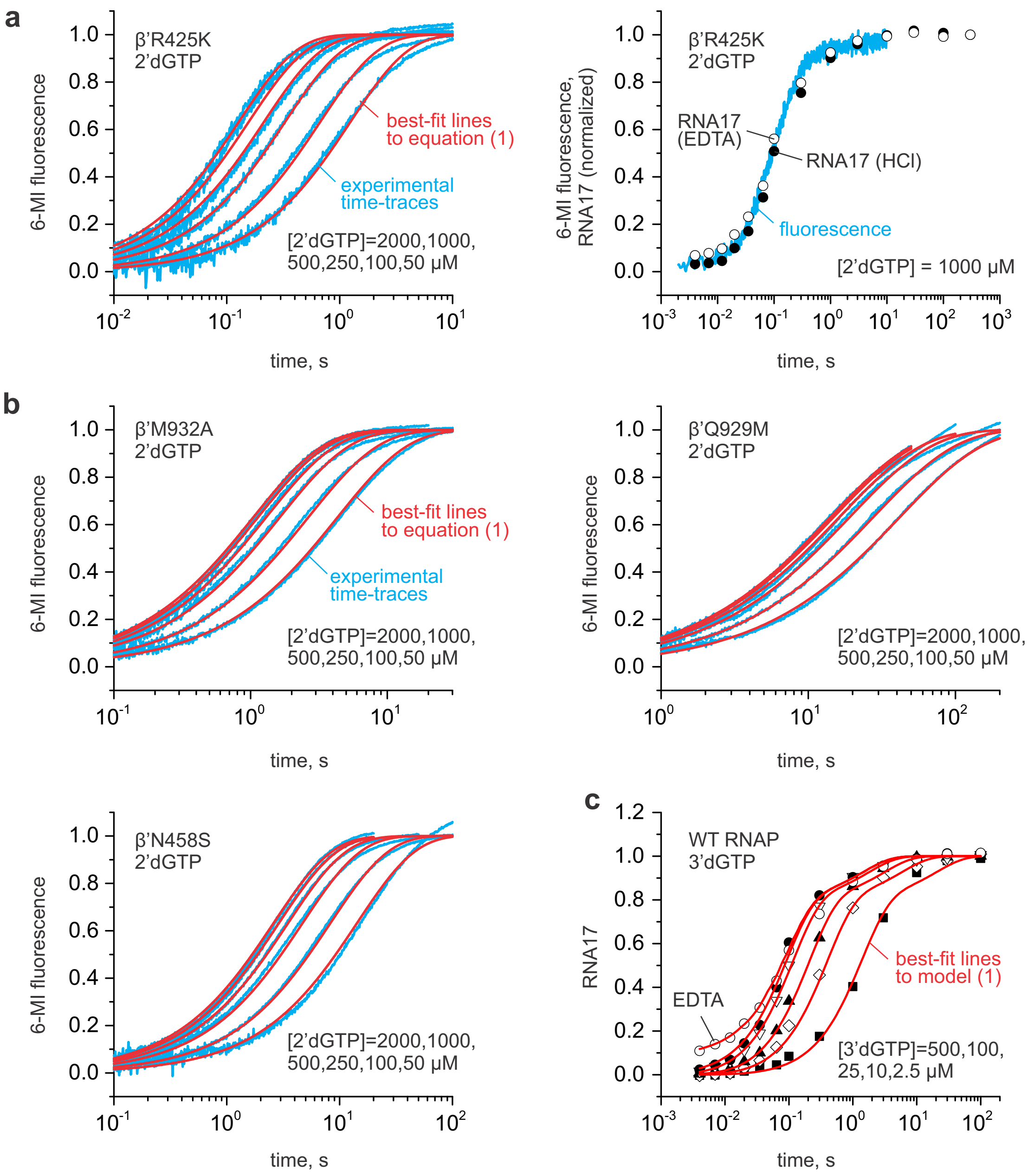

Supplementary Figure 2: 2'dGTP and 3'dGTP concentration series by the WT and variant *E. coli* RNAPs. a, **b** 2'dGMP concentration series were measured using the fluorescence assay and the data were fit to equation (1). The best-fit lines and fluorescence time-traces are colored red and cyan, respectively. The HCl and EDTA quenched data points are shown as closed and opened circles, respectively. **c** 3'dGTP concentration series were measured using the nucleotide addition assay and the data were fit to model (1). RNA extension time curves were obtained by HCl quench at five 3'dGTP concentration (closed circles, triangles, diamonds and squares). RNA extension time curve at 500 μM 3'dGTP was additionally measured using EDTA quench (open circles). All experiments were performed in duplicate, duplicate data were combined for the analysis. Source data are provided as a Source Data file.

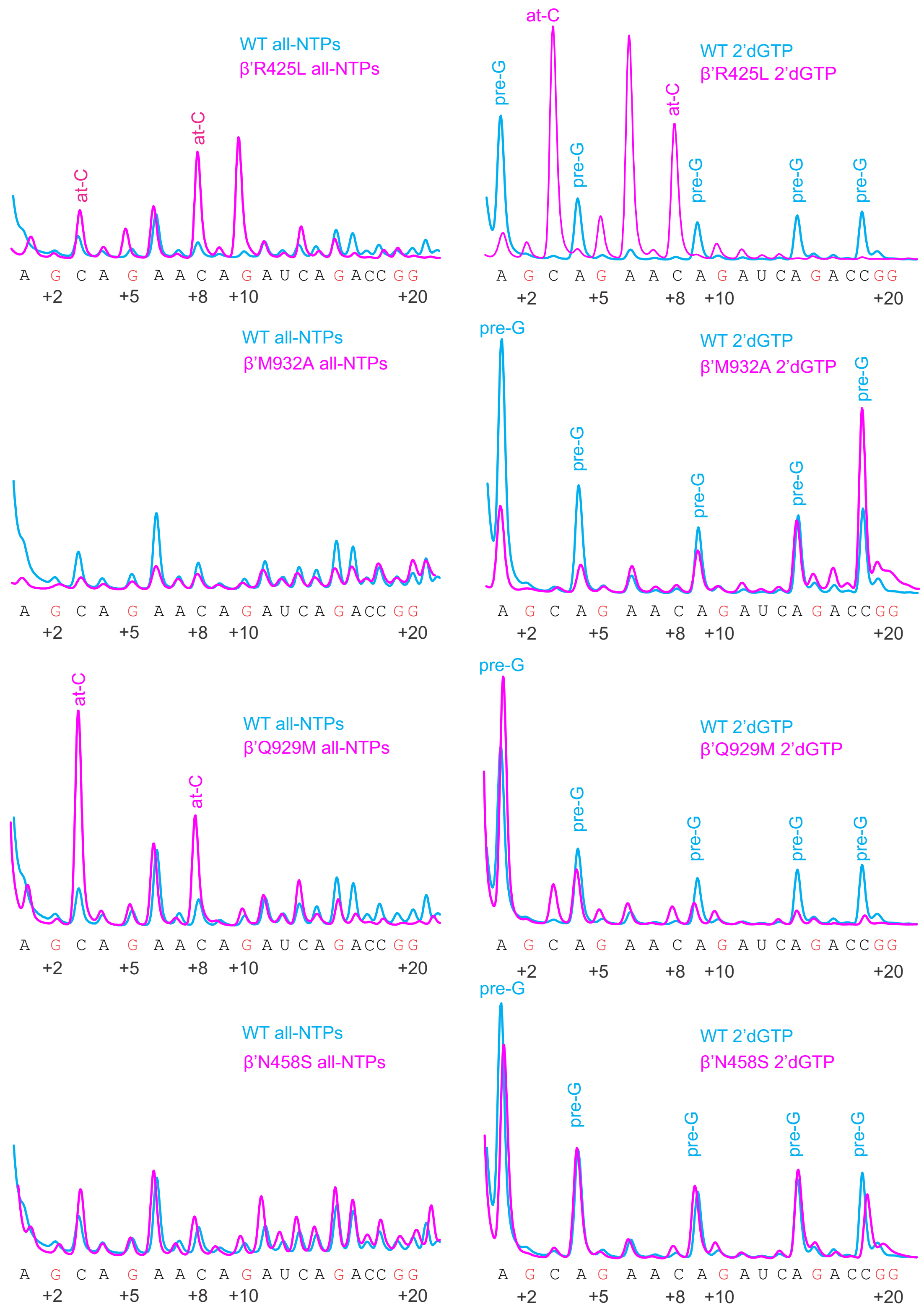

Supplementary Figure 3: Lane profiles of transcription in all-NTPs and 2'dGTP chases quantified from gels in main text Fig. 4. The WT and variant traces are colored cyan and magenta, respectively. All experiments were repeated in triplicate with similar results.

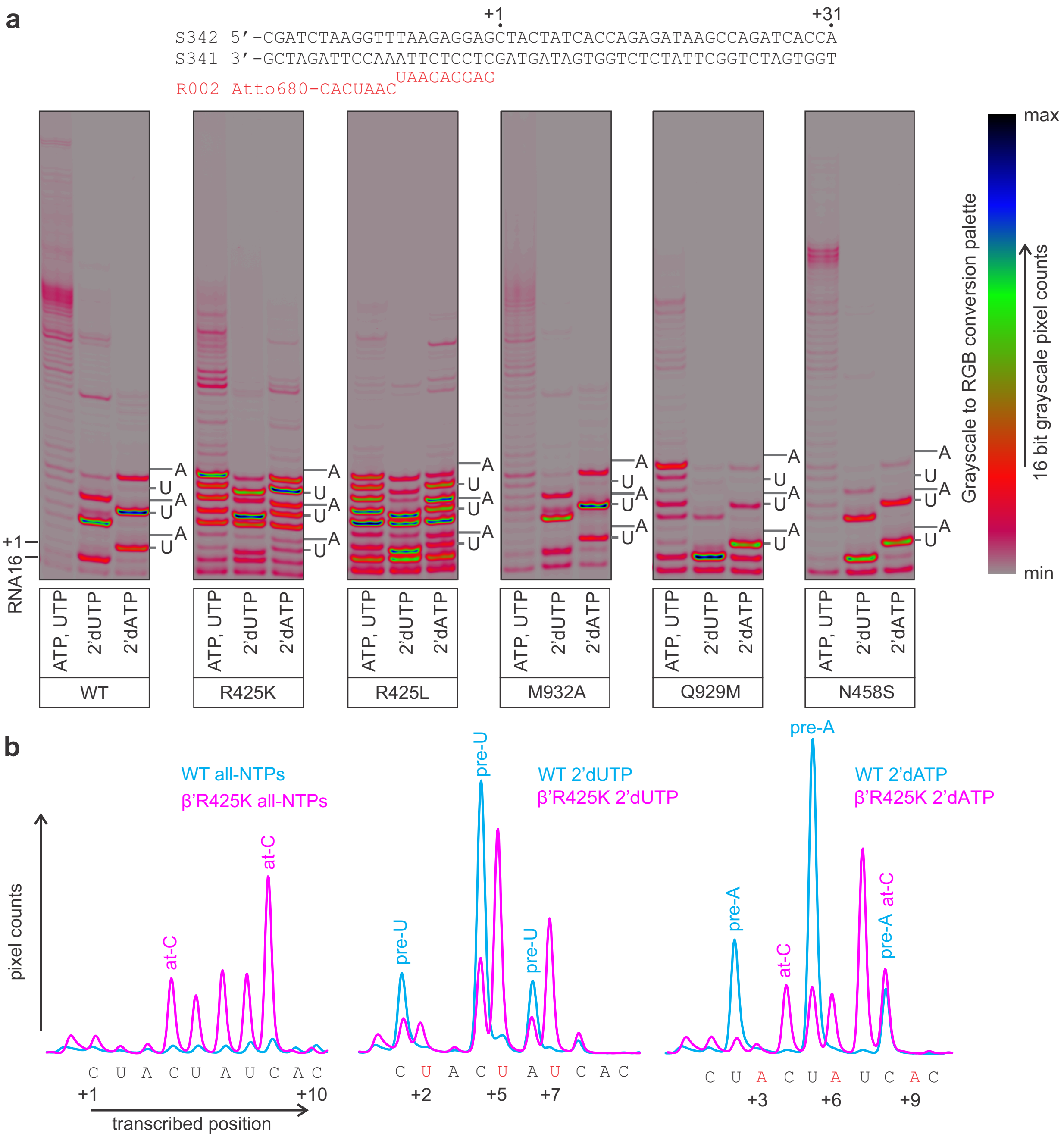

Supplementary Figure 4: Utilization of 2'dUTP and 2'dATP during processive transcript elongation by the WT and variant RNAPs. a TECs were assembled using the scaffold shown above the gel panels and chased with 100 µM CTP, GTP, UTP, ATP (all-NTPs-chase), or CTP, GTP, ATP, 2'dUTP (2'dUTP-chase), or CTP, GTP, UTP, 2'dATP (2'dATP-chase) for 5 min at 25°C. The positions of UMPs or AMPs in resolved stretches of the transcribed sequence are marked along the right edge of gel panels. 16-bit grayscale scans were normalized using highest pixel counts within each gel panel and pseudocolored using RGB palette. **b** Lane profiles of transcription by the WT (cyan) and β'R425K (magenta) RNAPs quantified from gels in **(a)**. Traces were manually aligned along the X-axis and scaled along the Y-axis using several sequence positions as references. All experiments were repeated in triplicate with similar results. Source data are provided as a Source Data file.

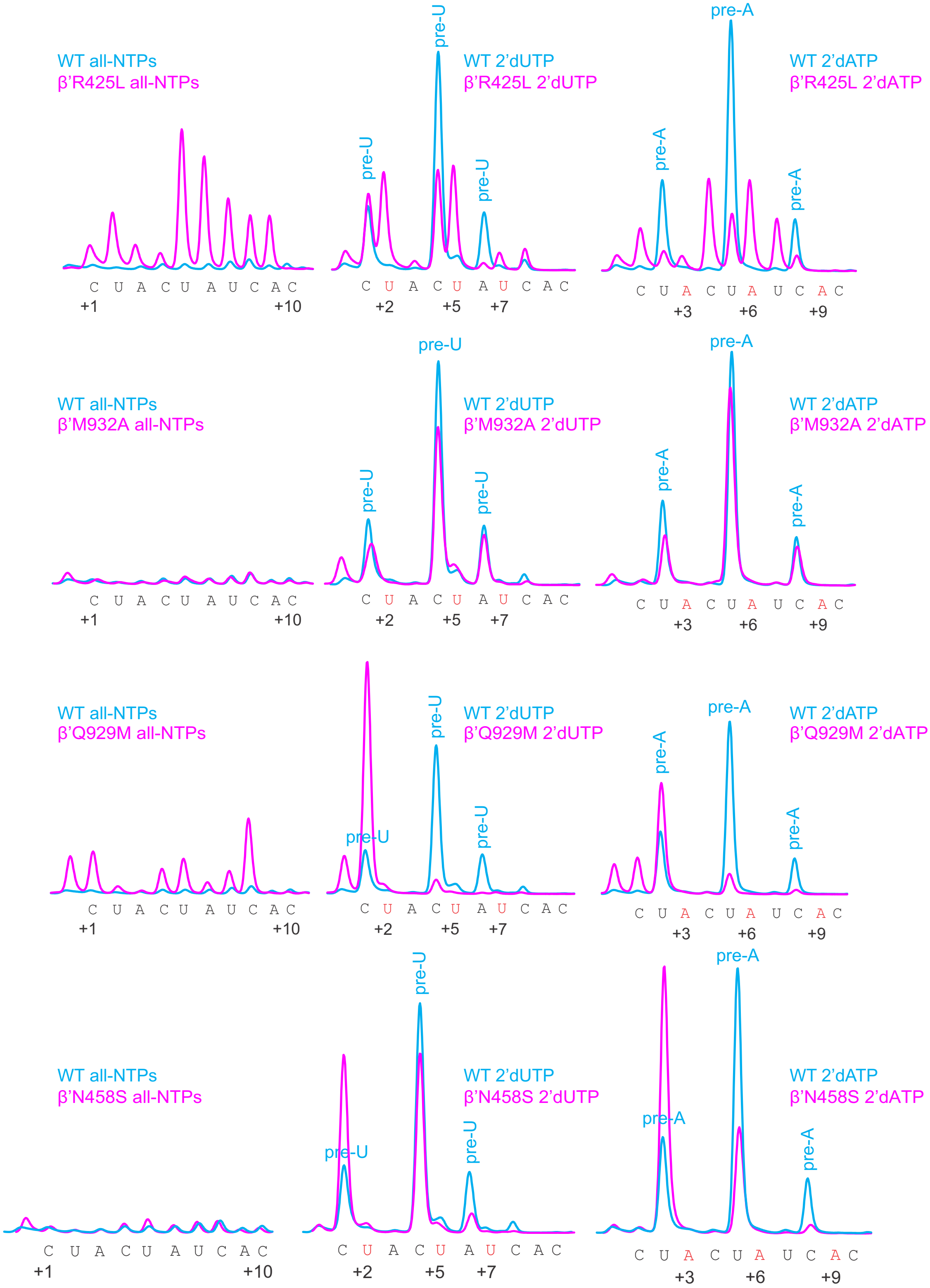

Supplementary Figure 5: Lane profiles of transcription in all-NTPs, 2'dUTP and 2'dATP chases quantified from gels shown in Supplementary Figure 4. The WT and variant traces are colored cyan and magenta, respectively. All experiments were repeated in triplicate with similar results.

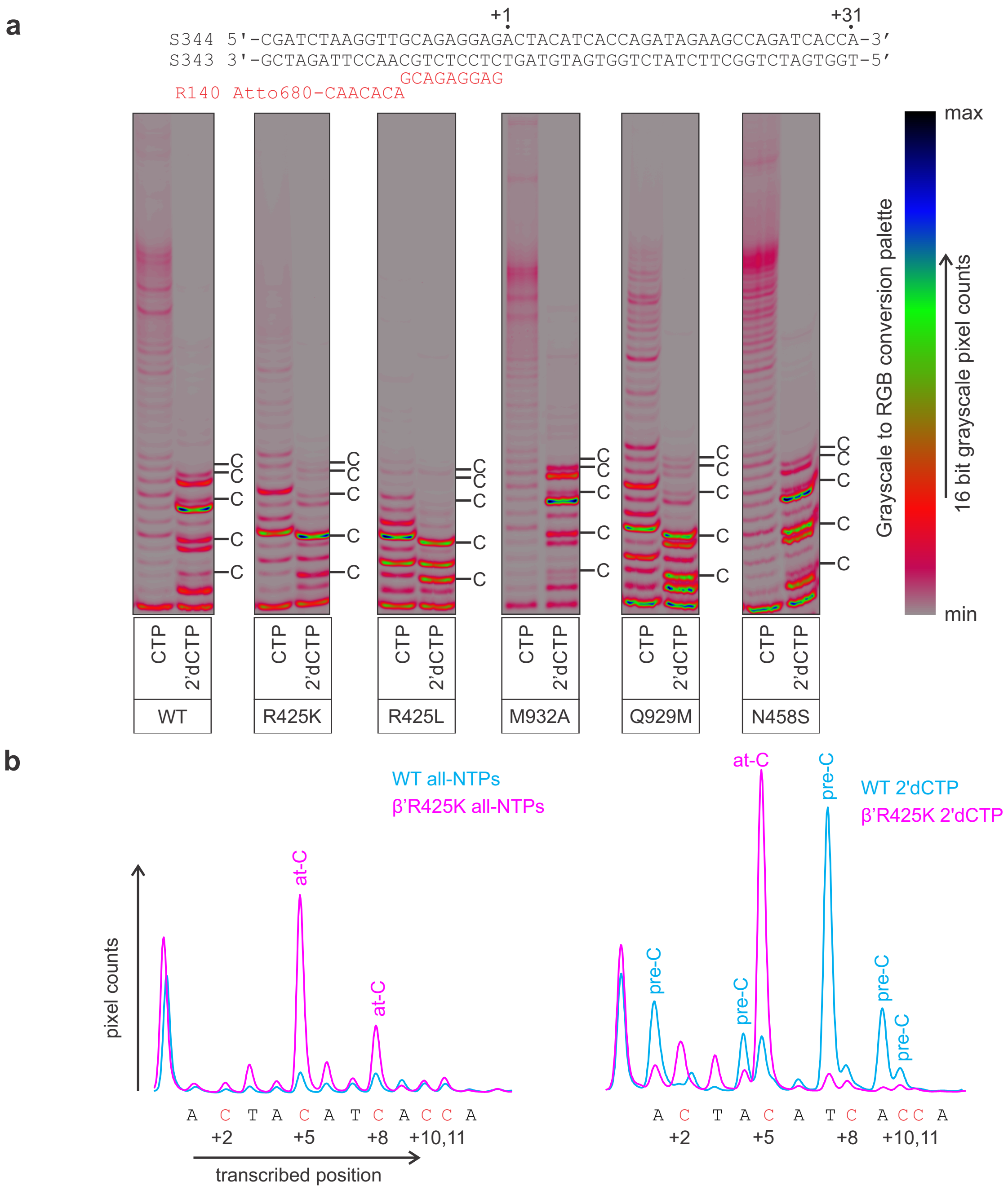

Supplementary Figure 6: Utilization of 2'dCTP during processive transcript elongation by the WT and variant RNAPs. a TECs were assembled using the scaffold shown above gel panels and chased with 100 µM GTP, UTP, ATP and CTP (all-NTPs chase) or 2'dCTP (2'dCTP chase) for 2 min at 25°C. **b** Lane profiles of transcription by the WT (cyan) and β'R425K (magenta) RNAPs quantified from gels in **(a)**. RNAPs with low K_D^{CTP} scavenged trace amounts of CTP when transcribing the first CMP encoding position in 2'dCTP-chase: the WT RNAP produced almost exclusively a slow migrating CMP extended RNA, whereas β'M932A produced a double band. RNAPs depleted CTP at the first position and incorporated exclusively 2'dCMP when transcribing CMP encoding positions further downstream. All experiments were repeated in triplicate with similar results. Source data are provided as a Source Data file.

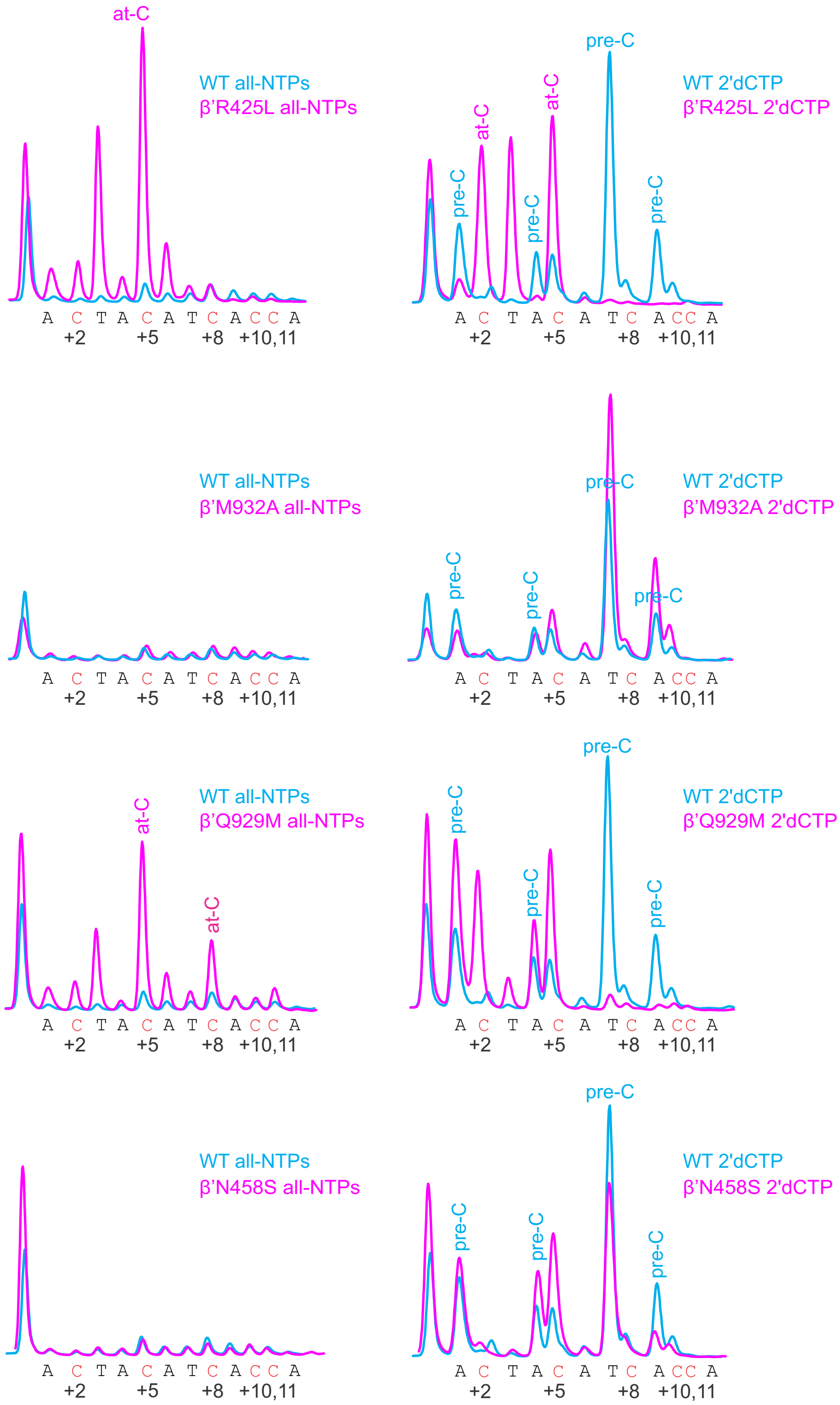

Supplementary Figure 7: Lane profiles of transcription in all-NTPs and 2'dCTP chases quantified from gels shown in Supplementary Figure 6. The WT and variant traces are colored cyan and magenta, respectively. All experiments were repeated in triplicate with similar results.

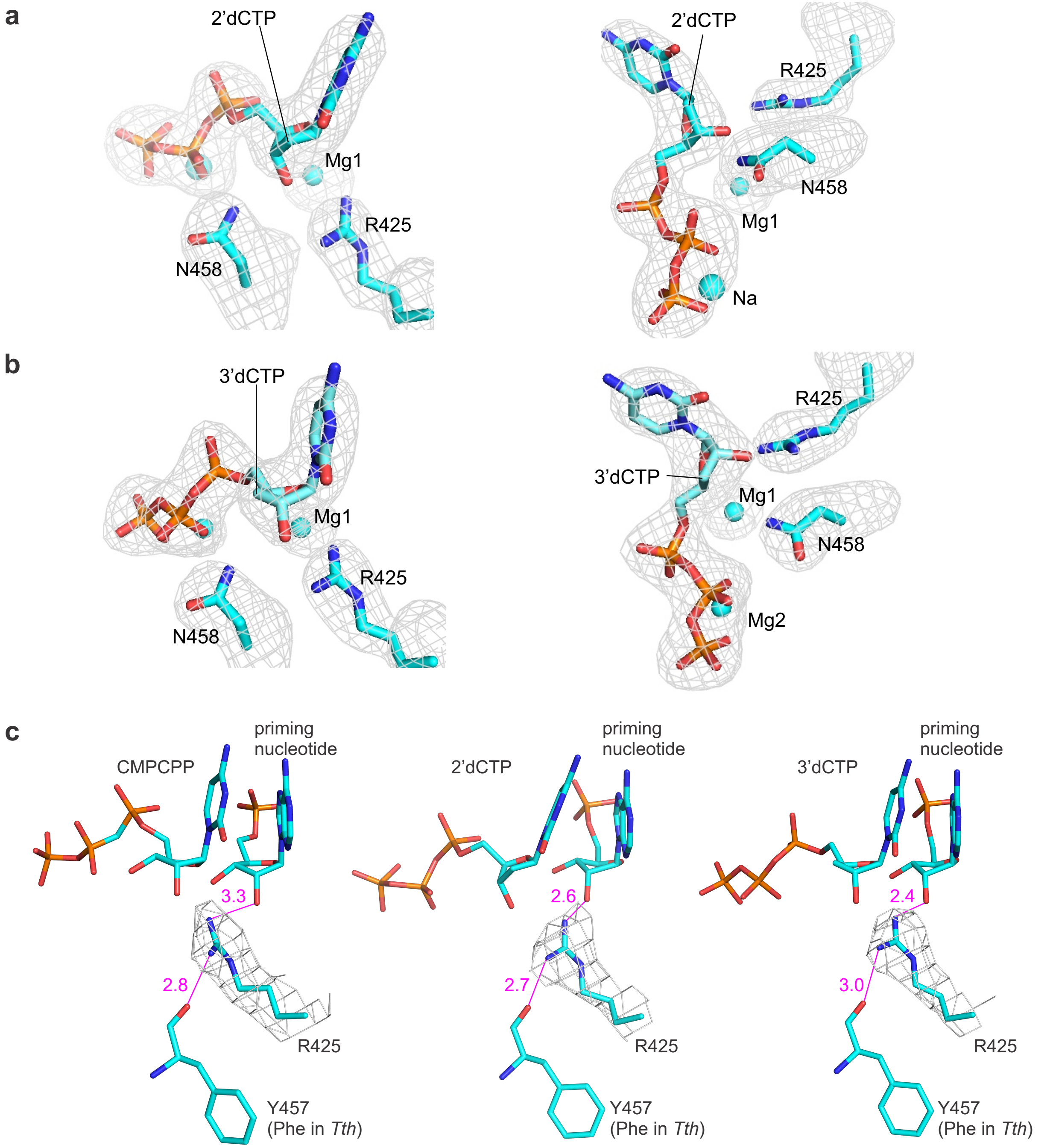

Supplementary Figure 8: Crystallographically observed binding poses of 2'dCTP and 3'dCTP in the active site of *T. thermophilus* RNAP. a, **b** Orthogonal views of 2Fo-Fc electron density maps (gray mesh, 1.0 σ) for the active site regions with bound 2’dCTP (**a**) and 3’dCTP (**b**). Backgrounds residues were removed for the clarity. 2’dCTP, 3’dCTP and amino acid residues β’Arg425 and β’Asn458 are shown as sticks. Mg^{2+} and Na^{+} ions are shown as cyan spheres. **c** β’Arg425 bridges the 2’OH of the priming nucleotide and the main chain carbonyl oxygen of β’Tyr457 in structures with ribo- (left), 2’deoxyribo- (center) and 3’deoxyribo- (right) nucleoside ligands. Magenta numbers are interatomic distances in Å. 2Fo-Fc electron density map for β’Arg425 is shown as a gray mesh (1.0 σ).

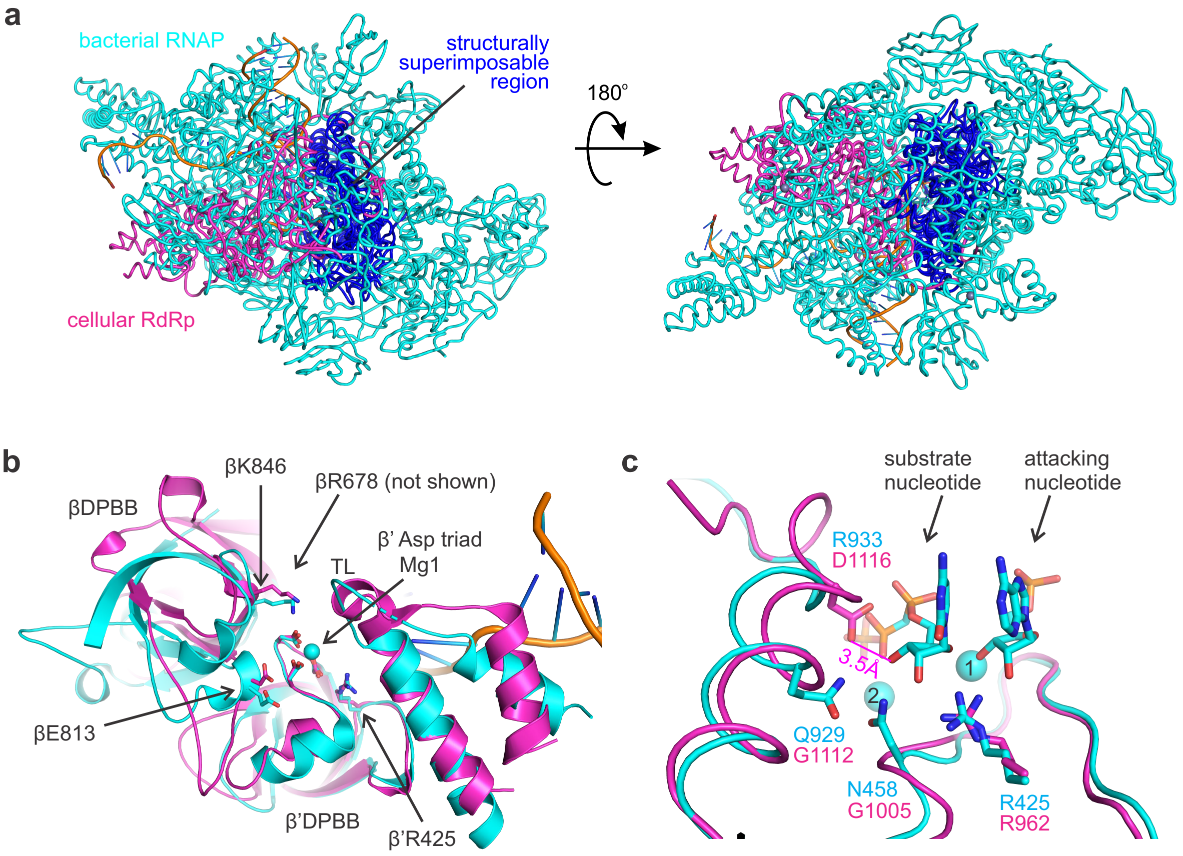

Supplementary Figure 9: Conservation of the nucleo-sugar recognition in two-β-barrel RNAPs. a A superimposition of a multi-subunit RNAP (cyan, PDB ID 4Q4Z) and a monomer of the homodimeric cellular RdRp (magenta, PDB ID 2J7N). Homologous regions are colored blue in both RNAPs. Structures were superimposed using the β'Arg425 and three Asp triad residues as references. **b** A close-up view of the active site. Six out of seven conserved catalytic residues (shown as sticks) are contributed by loops of the double-psi β-barrels (DPBB). In multi-subunit RNAPs, βDPBB and β'DPBB are contributed by β and β' subunits, respectively, whereas in RdRps both DPBBs belong to the same polypeptide chain. **c** A close-up view centered on the ribose moiety of the substrate. Catalytic Mg^{2+} ions are numbered. β'Arg425 adopts similar conformations in both RNAPs. In contrast, β'Asn458 and β'Gln929 correspond to Gly residues in cellular RdRps. Asp1116 of RdRp (corresponds to β'Arg933 in *E. coli* RNAP and Asn1082 in *S. cerevisiae* RNAPII) resides 3.5 Å from the 3'OH of the substrate from the superimposed structure. Asp1116 may therefore play a role in the recognition of the 3'OH of NTPs by RdRps.

Supplementary Note

Kinetic data analyses

We used time-resolved single nucleotide addition experiments to estimate the equilibrium constant for GTP, 2′dGTP and 3′dGTP binding and dissociation in the active site of RNAP and to determine the first order rate constant (also known as the turnover number) for the incorporation of GMP, 2′dGMP and 3′dGMP into the nascent RNA. The TECs were assembled on synthetic nucleic acid scaffolds and contained the fully complementary transcription bubble flanked by 20-nucleotide DNA duplexes upstream and downstream (Fig. 2a). The annealing region of a 16-nucleotide RNA primer was initially 9 nucleotides, permitting the TEC extended by one nucleotide to adopt the post- and pre-translocated states, but disfavoring backtracking. The RNA primer was 5′ labeled with the infrared fluorophore ATTO680 to monitor the RNA extension by denaturing PAGE.

To facilitate the rapid acquisition of kinetic data (see below), the template DNA strand contained a fluorescent base analogue 6-methyl-isoxanthopterin (6-MI) eight nucleotides upstream from the RNA 3′ end. 6-MI allowed the monitoring of RNAP translocation along the DNA following nucleotide incorporation (Fig. 2a). 6-MI was initially positioned in the RNA:DNA hybrid eight nucleotides upstream of the RNA 3′end. The 6-MI fluorescence was quenched by the neighboring base pairs in the initial TEC and the pre-translocated TEC that formed following nucleotide incorporation, but increased when the 6-MI relocated to the edge of the RNA:DNA hybrid upon translocation. This fluorescence system was extensively validated in our previous studies [28,29,56].

We first measured concentration series of GMP and 2′dGMP incorporation by the WT and altered RNAPs using a time-resolved fluorescence assay performed in a stopped flow instrument (Fig. 2, Supplementary Figures 1-2). We used the translocation assay because it allowed the rapid acquisition of concentration series, whereas measurements of concentration series by monitoring RNA extension in a rapid chemical quench-flow setup would be considerably more laborious. We then performed a preliminary data analysis by fitting each fluorescence timetrace to a single exponential function followed by fitting the resulting

individual rates to a Michaelis equation. The inferred k_{cat} and Km generally supported all major conclusions reported in this study. However, we proceeded to expand the datasets by including additional data and developed more elaborate analysis routines. The first reason to invoke a more elaborate analysis was the observation that most fluorescence time traces in our datasets fitted poorly to the single exponential function. In fact, the underlying physics of a single turnover enzymatic reaction suggests that individual timetraces in the concentration series should, in a general case, be poorly described by a single exponential function (see below). The second reason to invoke a more elaborate analysis was the concern that the Michaelis constant is a lumped constant that contains a sum of the catalytic and substrate dissociation rates in the numerator and the substrate binding rate in the denominator, whereas the equilibrium binding constants are the ratios of the substrate dissociation and binding rates. Accordingly, we were concerned that comparing the Michaelis constants of reactions could potentially lead to erroneous conclusions in the cases where the Km was markedly different from the K_D.

For the sake of understanding our analysis workflow, it is important to acknowledge that each reaction timetrace in the concentration series describes a single turnover process: we designed the transcribed sequence so that only a single GMP (or 2'dGMP or 3'dGMP) became incorporated upon the addition of GTP (or 2'dGTP or 3'dGTP). The ease of obtaining single turnover timetraces is a significant analytical advantage natively associated with template-dependent nucleic acid polymerases. It is often possible to infer more parameters from concentration series of single-turnover reactions than from concentration series of classic multi-turnover enzymatic reactions.

Next, most timetraces in the concentration series are not expected to fit a single exponential function even in the case of the simple signal, a 1-nt extended nascent RNA (RNA17 in this study). The enzymatic reaction is minimally a two-step sequential reaction that consists of the substrate binding and the substrate incorporation steps. For timetraces obtained at sub-saturating [NTP], both reactions are partially rate limiting and the overall shape of the timetrace cannot be accurately described by a single exponential function. Only timetraces at saturating [NTP] ($>10 \times Km$) and at the [NTP] far below saturation ($<Km/10$) are expected to fit well to a

single exponential function (for RNA17). Furthermore, fluorescence timetraces do not fit the single exponential function even at the saturating [NTP], because the process leading to the change in the fluorescence signal consists of three steps: NTP binding, NMP incorporation and translocation. Both the NMP incorporation and translocation are partially rate limiting at saturating [NTP] [29] resulting in the time-traces not following a single exponential function. The considerations above indicate that the concentration dependencies should be fit globally to the two step (RNA17) or three step (fluorescence timetraces) sequential reaction model (model (1)) rather than be fit individually to a single exponential function.

We fitted the GTP, 3'dGTP and selected 2'dGTP concentration series to model (1) using the numerical integration capabilities of the Kintek Explorer software [44]. The model postulated that the initial TEC16 reversibly binds the NTP substrate, undergoes an irreversible transition to TEC17 upon incorporation of the nucleotide into RNA, followed by an irreversible translocation. The concentration series data (fluorescence timetraces supplemented with a single HCl-quenched RNA extension curve at a saturating substrate concentration) allowed for the inference of the k_{cat}, K_D and k_{tra} (in some cases), whereas the individual values of the k_{on} and k_{off} could not be resolved. Incorporation of an EDTA quench experiment allowed for the resolution of all parameters in model (1) in cases where the EDTA quenched curve was temporarily separated from the HCl quench curve (Table 1, GTP and 3'dGTP data; Supplementary Table 3). In cases where the EDTA quenched curve was not temporarily separated from the HCl quench curve, only the lower bounds of the k_{on} and k_{off} could be inferred (Table 1, 2d'GTP data). Overall, fits to model (1) were performed largely as described in Prajapati et al. [45] except that here we used a non-equilibrium heterogeneity model (see below) to describe the kinetic heterogeneity in the TEC preparations instead of a reversible inactivation model (see below) that was employed by Prajapati et al.

Next, a kinetic heterogeneity in the TEC preparations introduced an additional level of complexity to the fitting of the data. We reported previously that a vast majority of TECs contain 5-20% of a slow fraction that manifests itself as a slow phase in reaction timetraces of both the fluorescence signal (stopped-flow assay) and the extended RNA (quench flow assay)

[29,56]. In the case of fast reactions measured in this study (GTP, 3′dGTP data), the rates of the fast and slow phases differed approximately tenfold and therefore the phases could be precisely resolved (see a dedicated section below). Importantly, the fast phase of the reaction constituted 80-90% of the signal amplitude (Table 1, Supplementary Table 3). Accordingly, we considered the activity of the fast fraction as a representative measure of the RNAP activity in each experiment and disregarded the minor slow fraction when comparing the WT and variant RNAPs (Fig. 3).

In the case of slow reactions (2′dGTP data), the fast and the slow phases were not well separated (4-fold difference in rates, Table 1, 2′dGTP data for the WT RNAP). The poor separation led to large uncertainties in the k_{cat} and the percentage of the fast fraction. Moreover, the fitting algorithm partitioned the signal amplitude approximately equally between the fast and the slow phases so the activity of the fast fraction could not be used as a representative measure of the RNAP activity in each experiment (Table 1, 2′dGTP data for the WT RNAP). To circumvent this problem, we globally fit the 2′dGTP concentration series to a semi-empirical equation (1) instead of using model (1).

When fitting data to equation (1), each timetrace was described by a stretched exponential function (an empirical function that is often used to describe heterogeneous systems Flomenbom, O. *et al*. (2005) *Proc. Natl. Acad. Sci. USA* 102, 2368–2372). At the same time, the exponent followed the hyperbolic dependence on the 2′dGTP concentration (Fig. 2c, Supplementary Figure 2). Such fits described the data well and gave three parameters: a reaction rate constant (k), a stretching parameter (β) and the Michaelis constant (Km). When a stretching exponential function is applied to a process where the reactivity changes over time (or distance), the rate constant parameter (k) corresponds to the initial reaction rate constant. In our case, the stretched exponential fit potentially absorbed both temporal and structural heterogeneity as well as the deviations from the single exponential behavior caused by the sequential nature of the enzymatic reaction (see above). For this reason, the rate parameter (k) did not have an easily interpretable meaning. To circumvent this problem we calculated the median reaction time as (median reaction time) = $(\ln(2)^{(1/\beta)}) / k$; then calculated the median

reaction rate assuming that (median reaction rate) = ln(2) / (median reaction time) and used the median reaction rate as a measure when comparing the WT and variant RNAPs (Fig. 3, Table 2). Next, fitting the data to equation (1) gives the Km rather than the K_D. However, it is rather certain that $k_{off} >> k_{cat}$ for all 2′dGMP incorporation reactions (Table 1, also see Scenario 2 below). If so, Km approximately equals K_D for each 2′dGMP incorporation reaction. Accordingly, we used Km in place of K_D for 2′dGMP addition reactions when comparing substrates and RNAPs (Fig. 3).

Finally, we emphasize that the 2′dGMP incorporation data by the WT and the β′R425K RNAPs were fit to both model (1) and equation (1) leading to affinities for 2′dGTP that were indistinguishable within the margin of the experimental uncertainty (compare 2′dGTP data in Table 1 and Table 2). The catalytic activity of the WT RNAP towards 2′dGTP inferred by fitting the data to equation (1) was, as expected, in-between the catalytic activities of the fast and slow fraction inferred by fitting the data to model (1). Accordingly, we argue that the employment of different analysis routines for GTP and 2′dGTP is of little concern for the main inferences drawn in this study.

Handling of the translocation rate during the kinetic analysis of nucleotide binding and incorporation

We have previously shown that the nucleotide addition and the subsequent translocation along the DNA by the WT *E. coli* RNAP occur with similar rates at saturating concentrations of cognate NTPs [29]. As a result, (*i*) the translocation timetraces are delayed by a few milliseconds relative to the nucleotide addition timecurves and (*ii*) the translocation timetraces at saturating concentrations of cognate NTP substrates are not well described by a single exponential function because both nucleotide addition and translocation are partially rate limiting. In this study, translocation rates were tangential to the main line of investigation, but they were necessary parameters during the global fitting of the fluorescence timetraces and GMP incorporation timecurves to model (1). At the same time, the translocation rates are much faster than the 2′dGMP incorporation rates and could be completely disregarded during the analysis of the 2′dGTP concentration series by fitting the date to model (1) or equation (1).

It should be noted that translocation rates reported in Supplementary Table 3 should not be equated with the forward translocation rates. Thus, we modeled translocation as an irreversible transition in model (1). As a result, the inferred translocation rates are the rates of the system approaching the translocation equilibrium after the nucleotide incorporation rather than the forward translocation rates. Albeit somewhat counterintuitively but following the rules of the formal kinetics the inferred equilibration rate equals the sum of the forward and the backward translocation rates. It was possible to further split the equilibration rate into the forward and backward translocation rates by assessing the completeness of the translocation, as we did in our previous studies [56]. However, we refrained from doing so in this study because the translocation process was tangential to the main line of the investigation.

Interpretation of EDTA quench experiments

EDTA inactivates the free NTPs by chelating Mg^{2+} but allows a fraction of the NTPs that are already bound in the RNAP active site to complete incorporation into the RNA. In contrast, HCl denatures RNAP so neither free nor RNAP-bound NTPs can be incorporated into the RNA after the addition of HCl. As a result, a comparison of the EDTA-quenched timecurve and HCl-quenched timecurve may provide information about the NTP dissociation rate from the active site. If the rate of NTP dissociation from the active site is comparable to (or smaller than) the catalytic rate, the EDTA-quenched curve reports more extended RNA than the HCl-quenched curve at each timepoint [30]. In contrast, if the rate of NTP dissociation from the active site is much larger than the catalytic rate, the EDTA- and HCl-quenched curves superimpose. Consistently, Kireeva et al. showed that the EDTA quench experiment is fully equivalent to the pulse-chase setup when performed with the *S. cerevisiae* RNAPII [31]. We explicitly modeled the EDTA quench experiments using the pulse-chase routine of the Kintek explorer software. Such an approach does not require a priori assumptions about the reaction rates in the three-step model employed for fitting the data. However, we consider it imperative to recognize the graphic signatures of the EDTA-quenched curves, rather than solely rely on the fitting algorithm as a "black box" to estimate the parameters. Accordingly, we discuss the shape of the EDTA-quenched curve under three scenarios with different ratios of k_{cat} and k_{off} and relate them to our data.

Scenario 1. The rate of NTP dissociation from the active site (k_{off}) is much lower than the rate of NMP incorporation (k_{cat}). In this case, EDTA quench curves of a fully post-translocated and kinetically homogeneous TEC are expected to fit a single exponential function with the exponent corresponding to the pseudo first order rate constant for NTP binding (k_{on} × [NTP]) (Supplementary Note Fig. 1a). As a result, both TEC17 and TEC16NTP are detected as TEC17 in the EDTA quenched samples because nearly 100% of the TEC16NTP is converted into TEC17 after the addition of EDTA, and practically no NTP dissociates back into the solution ($k_{cat} >> k_{off}$). In this situation, EDTA quenched curves precede HCl quenched curves at saturating [NTP]: the HCl curve is limited by k_{cat}, whereas the EDTA curve is limited by k_{on} × [NTP]. In this scenario, at least k_{cat} and k_{on} can be inferred from NTP concentration series alone because $Km = (k_{cat} + k_{off})/k_{on} \approx k_{cat}/k_{on}$, so $k_{on} \approx k_{cat}/Km$. The global fit of the NTP concentration series and the EDTA quench data additionally allows the inference of the upper bounds of k_{off} and K_D.

We did not encounter Scenario 1 in this study though the β′M932A data are a borderline case that resembles Scenario 1. While both the upper and lower bounds of k_{off} could be determined (Supplementary Table 3) the inferred values were markedly smaller than the k_{cat} of 17 - 29 s^{-1} and the lower bound for k_{off} was therefore very diffuse: best fit 3.1 s^{-1}, lower bound 0.8 s^{-1}, upper bound 5.4 s^{-1}.

Scenario 2. The rate of NTP dissociation from the active site (k_{off}) is much faster than the rate of NMP incorporation (k_{cat}). In this case, only TEC17 is detected as TEC17 in the EDTA quenched samples, because nearly 100% of the TEC16NTP loses its NTP after the addition of EDTA and practically no NMP gets incorporated into the RNA after the addition of EDTA ($k_{cat} << k_{off}$). In this situation, EDTA quenched curves always superimpose with HCl quenched curves. At least k_{cat} and K_D can be estimated from the NTP concentration series alone because $Km = (k_{cat} + k_{off})/k_{on} \approx k_{off}/k_{on} = K_D$. The global fit of the NTP concentration series and the EDTA quench data additionally allow the inference of the lower bounds of k_{on} and k_{off}.

The above situation corresponds to 2′dGMP addition by the WT and variant RNAPs. Fitting the 2′dGTP concentration series to a semi-empirical equation (1) allowed the estimation of k_{cat} and $Km^{2'dGTP} \approx K_D^{2'dGTP}$ for the WT, β′R425K, β′M932A, β′Q929M and β′N458S RNAPs (Table 2). For

the β′R425K and the WT RNAP we additionally measured the EDTA quench curve, fitted the data globally to model (1) and inferred the lower bounds of k_{on} and k_{off}. in addition to K_D (Table 1, Fig. 2c, Supplementary Figure 2a).

Scenario 3. The rate of NTP dissociation from the active site (k_{off}) is similar to the rate of NMP incorporation (k_{cat}). In this case, EDTA quench curves of a fully post-translocated and kinetically homogeneous TEC obtained at saturating [NTP] are expected to fit a biexponential function with the first exponent corresponding to the pseudo first order rate constant of NTP binding (k_{on} × [NTP]) and the second exponent corresponding to k_{cat}. For normalized data, the amplitude of the first exponent equals $k_{cat}/(k_{cat} + k_{off})$ and the amplitude of the second exponent equals $k_{off}/(k_{cat} + k_{off})$. In this situation, EDTA-quenched curves precede HCl-quenched curves at saturating [NTP]: The HCl curve is limited by k_{cat}, whereas the fast phase of the EDTA curve is limited by k_{on} × [NTP]. As always, k_{cat} and Km can be inferred from the NTP concentration series, but neither $k_{cat}/Km \approx k_{on}$ (as is in Scenario 1) nor $Km \approx K_D$ (as is in Scenario 2). In contrast, the global fit of the NTP concentration series and the EDTA quench data has the best resolving power in Scenario 3: k_{cat}, k_{on}, k_{off}, and k_{tra} (in some cases) can be inferred from the data though the precision of the individual estimates varies greatly.

The above situation corresponds to the GMP addition by the WT and variant RNAPs (Supplementary Table 3, Fig. 2b, Supplementary Figure 1) and the 3′dGMP addition by the WT RNAP (Table 1, Supplementary Figure 2c). Only the WT RNAP data allowed for precise estimates of all parameters of model (1). In the case of the β′R425K and β′Q929M variants, the EDTA- and HCl-quenched curves separated poorly resulting in diffuse upper bounds for k_{on} and k_{off}. In the case of all variant RNAPs, the HCl-quenched curves and the fluorescence timetraces separated poorly resulting in diffuse upper bounds for k_{tra}. Finally, the EDTA-quenched curve of the β′M932A RNAP featured little change in the signal (RNA17) within the measured time interval resulting in a diffuse lower bound for k_{off}. With all that said, relatively precise estimates for k_{cat} and K_D were obtained for all RNAPs and used as measures for the comparison of the RNAP's capabilities to bind and utilize various substrates (Fig. 3).

Handling of the slow fraction during fitting to model (1). The time-courses of the NMP incorporation by the WT *E. coli* TEC typically display a distinctive slow phase that represents 5-25% of the overall signal amplitude and features the rate of 0.1 - 3 s^{-1}. In contrast, the major, fast phase of the reaction is approximately tenfold faster at saturating [NTP] (20 - 30 s^{-1} for GTP). The slow phase possibly represents an inactive TEC in equilibrium with the active TEC, a fraction of the TEC that slowly reacts with the NTP substrate or a combination of both. During the fitting of the data using the Kintek Explorer software, the slow phase can be modeled in two ways (Supplementary Note Fig. 1b). The first option is to invoke a reversible equilibrium between the active and inactive TEC and to introduce a virtual equilibration step prior to mixing of the TEC with the NTPs. We term this approach as the reversible inactivation model. The second option is to explicitly model the TEC preparation as two fractions that do not interconvert but incorporate NMP with different rates. The fractions of the slow and fast TEC are then allowed to vary as parameters during the fit. We term this approach as the non-equilibrium heterogeneity model.

The two models are largely indistinguishable if measurements are carried out at a single [NTP] and both models require two parameters to describe the slow phase: inactivation and recovery rates in the first case, and the slow fraction and its reaction rate in the second case (Supplementary Note Fig. 1b). However, the response of the slow phase to the decrease in the [NTP] differs between these two models. The reversible inactivation model predicts that the rate of the slow phase is independent of [NTP] and the slow phase is largely abolished as the [NTP] decreases. In contrast, the non-equilibrium heterogeneity model predicts that the rate of the slow phase decreases in unison with the rate of the fast phase as [NTP] decreases (both follow a hyperbolic dependence on [NTP]). In this study we analyzed all GMP and 3′dGMP incorporation datasets using the non-equilibrium heterogeneity approach to model the slow phase, because some datasets (e.g. β′Q929M, Supplementary Figure 1) could not be adequately fit by the previously employed reversible inactivation model [28,45,56].

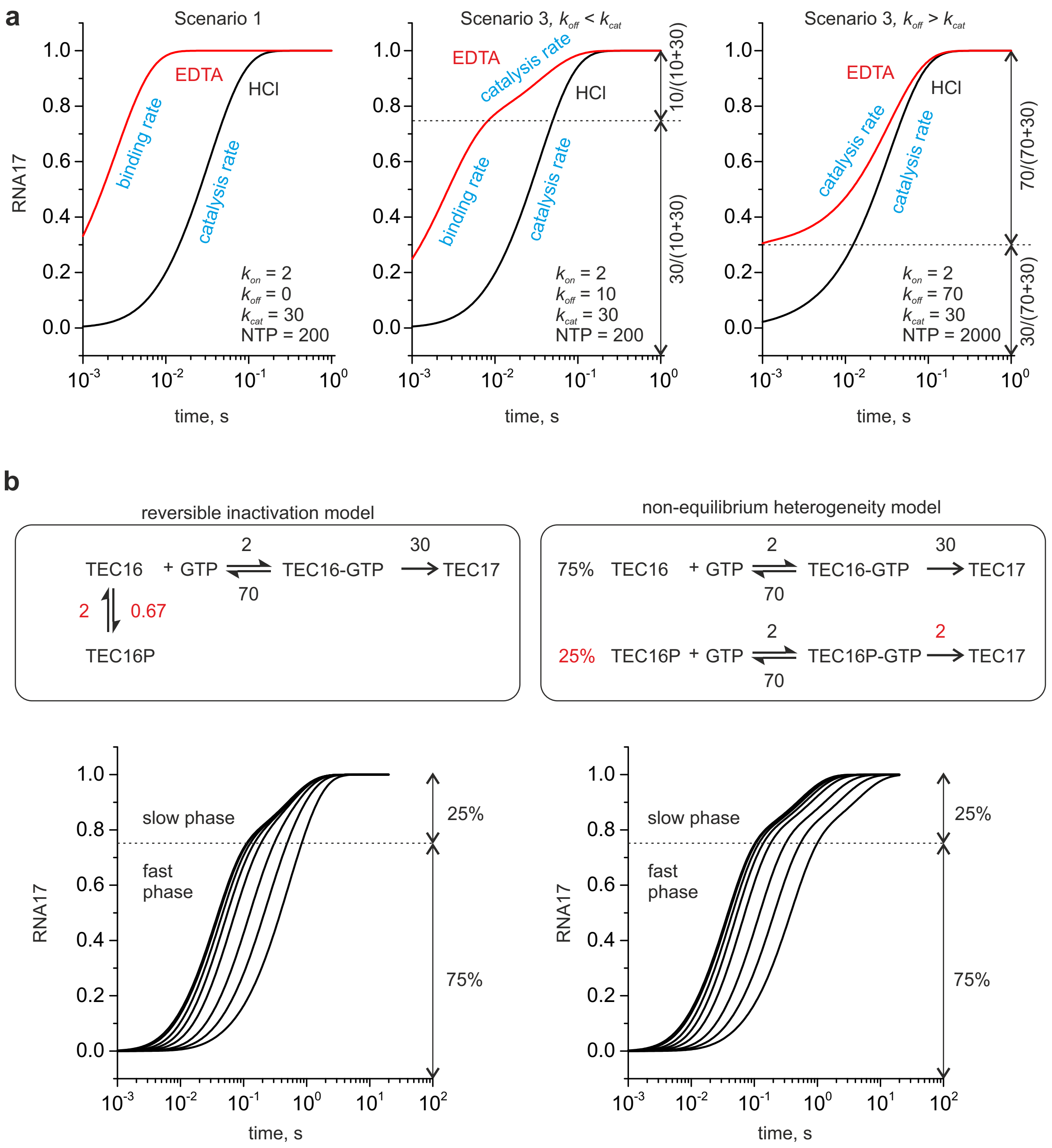

Supplementary Note Fig. 1: Kinetic analyses of the data. **a** Simulation and graphic interpretation of the EDTA and HCl quench curves at saturating substrate concentrations and different values of k_{off}. EDTA quench curves are colored red. **b** Simulation of concentration series of a biphasic reaction using the reversible inactivation (left) and non-equilibrium catalytic heterogeneity (right) models. Parameters that describe the slow phase are colored red.
