# Supplementary material for: The mechanism of the nucleo-sugar selection by multi-subunit RNA polymerases
Source: Nat Commun. 2021 Feb 4;12:796. doi: 10.1038/s41467-021-21005-w (PMC7862312; doi:10.1038/s41467-021-21005-w)
Supplement: Supplementary file 3 — Reporting Summary [file 41467_2021_21005_MOESM3_ESM.pdf]

nature research

Corresponding author(s): Katsuhiko Murakami, Georgiy Belogurov

Last updated by author(s): Jan 4, 2021

Reporting Summary

Nature Research wishes to improve the reproducibility of the work that we publish. This form provides structure for consistency and transparency in reporting. For further information on Nature Research policies, see Authors & Referees and the Editorial Policy Checklist .

Statistics

For all statistical analyses, confirm that the following items are present in the figure legend, table legend, main text, or Methods section.

n/a	Confirmed	
☐	☒	The exact sample size (*n*) for each experimental group/condition, given as a discrete number and unit of measurement
☐	☒	A statement on whether measurements were taken from distinct samples or whether the same sample was measured repeatedly
☐	☒	The statistical test(s) used AND whether they are one- or two-sided<br>*Only common tests should be described solely by name; describe more complex techniques in the Methods section.*
☐	☒	A description of all covariates tested
☐	☒	A description of any assumptions or corrections, such as tests of normality and adjustment for multiple comparisons
☐	☒	A full description of the statistical parameters including central tendency (e.g. means) or other basic estimates (e.g. regression coefficient) AND variation (e.g. standard deviation) or associated estimates of uncertainty (e.g. confidence intervals)
☒	☐	For null hypothesis testing, the test statistic (e.g. *F*, *t*, *r*) with confidence intervals, effect sizes, degrees of freedom and *P* value noted<br>*Give P values as exact values whenever suitable.*
☒	☐	For Bayesian analysis, information on the choice of priors and Markov chain Monte Carlo settings
☒	☐	For hierarchical and complex designs, identification of the appropriate level for tests and full reporting of outcomes
☒	☐	Estimates of effect sizes (e.g. Cohen's *d*, Pearson's *r*), indicating how they were calculated

Our web collection on statistics for biologists contains articles on many of the points above.

Software and code

Policy information about availability of computer code

Data collection	ADX software vCHESS2015
Data analysis	HKL2000 v714, Coot v0.8.9.2, Phenix v1.15.2-3472, Origin 2015 Sr2 b9.2.272, Kintek Explorer 6.3.180116, ImageJ 1.52a, PyMol v1.7.4.1, PyMOL v2.3.3.

For manuscripts utilizing custom algorithms or software that are central to the research but not yet described in published literature, software must be made available to editors/reviewers. We strongly encourage code deposition in a community repository (e.g. GitHub). See the Nature Research guidelines for submitting code & software for further information.

Data

Policy information about availability of data

All manuscripts must include a data availability statement. This statement should provide the following information, where applicable:

- Accession codes, unique identifiers, or web links for publicly available datasets
- A list of figures that have associated raw data
- A description of any restrictions on data availability

X-ray crystallographic structure coordinates and their structural factors have been deposited in the RCSB Protein Data Bank (https://www.rcsb.org/) with accession numbers 6WOX [https://www.rcsb.org/structure/6WOX] and 6WOY [https://www.rcsb.org/structure/6WOY]. Source data for Figs. 2, 4, 5, 6, 7 and Supplementary Figures 1, 2, 4, 6 are provided. Other data are available from the corresponding authors upon reasonable request.

Field-specific reporting

Please select the one below that is the best fit for your research. If you are not sure, read the appropriate sections before making your selection.

☒ Life sciences ☐ Behavioural & social sciences ☐ Ecological, evolutionary & environmental sciences

For a reference copy of the document with all sections, see nature.com/documents/nr-reporting-summary-flat.pdf

Life sciences study design

All studies must disclose on these points even when the disclosure is negative.

Sample size

This study reports results of in vitro biochemical and in silico docking experiments and X-ray crystallography, no biological samples were collected. The inferred kinetic parameters were independent on the specific activity of enzymes in preparations, because all transcription experiments were performed in single round mode. Three different single nucleotide addition assays were performed 2 times each (3 assays, n=2 independent experiments each) and duplicate data from all three assays were analyzed globally. The upper and lower bounds of the kinetic parameters were calculated by FitSpace routine of the Kintek Explorer software or by Origin 2015 software. These upper and lower limits define ranges of the parameters values that support a good fit of the model to data (≤ 10% increase in Chi2) and are not measures of the biological variability. Conclusions were based on 10 to 20-fold differences in kinetic parameters. P-value testing and the determination of the sample size were not required and not performed. Processive transcription assays were performed 3 times (n=3 independent experiment). Conclusions were based on 2-3 fold differences in intensities of selected bands. P-value testing and the determination of the sample size were not required and not performed. In silico docking experiments were performed 10 times (n = 10 independent runs). The calculated 95% confidence intervals of pose recovery frequencies suggested that poses were robustly recovered in ligand-receptor pairs that we aimed to compare. The calculated 95% confidence intervals of the binding scores suggested that the average binding scores were similar in ligand-receptor pairs that we aimed to compare.

Data exclusions

Bad X-ray diffraction data marked for rejection during the scaling process by HKL2000 in the process of structure determination of PDB IDs 6WOX and 6WOY were discarded.

Replication

All single nucleotide addition assays were performed at least two times using independently assembled transcription elongation complexes. All processive transcription assays were performed at least three times using independently assembled transcription elongation complexes. During in silico docking experiments at least ten independent docking runs were performed. All results were reproducible.

Randomization

This study reports results from rationally designed in vitro biochemical/biophysical experiments, for which randomization is not applicable, as there is no danger of confounding independent variables in the experimental design.

Blinding

This study reports results from rationally designed in vitro biochemical/biophysical experiments, for which blinding is not applicable, as the experiments did not involve human subjects, and as the results from the experiments can be objectively evaluated/quantified.

Reporting for specific materials, systems and methods

We require information from authors about some types of materials, experimental systems and methods used in many studies. Here, indicate whether each material, system or method listed is relevant to your study. If you are not sure if a list item applies to your research, read the appropriate section before selecting a response.

Materials & experimental systems

n/a	Involved in the study	
☒	☐	Antibodies
☒	☐	Eukaryotic cell lines
☒	☐	Palaeontology
☒	☐	Animals and other organisms
☒	☐	Human research participants
☒	☐	Clinical data

Methods

n/a	Involved in the study	
☒	☐	ChIP-seq
☒	☐	Flow cytometry
☒	☐	MRI-based neuroimaging
