# Supplementary material for: The mechanism of the nucleo-sugar selection by multi-subunit RNA polymerases
Source: Nat Commun. 2021 Feb 4;12:796. doi: 10.1038/s41467-021-21005-w (PMC7862312; doi:10.1038/s41467-021-21005-w)
Supplement: Supplementary file 4 — Source Data [file 41467_2021_21005_MOESM4_ESM.zip › 3D_models_Fig_5_6_7/Fig_5c.pdf]

Rotate

Zoom

Pan

In silico docking of the 3'-endo 2'dCMP in the active site of *T. thermophilus* RNAP.

Two representative poses are colored cyan and magenta.
