# Supplementary material for: The mechanism of the nucleo-sugar selection by multi-subunit RNA polymerases
Source: Nat Commun. 2021 Feb 4;12:796. doi: 10.1038/s41467-021-21005-w (PMC7862312; doi:10.1038/s41467-021-21005-w)
Supplement: Supplementary file 4 — Source Data [file 41467_2021_21005_MOESM4_ESM.zip › 3D_models_Fig_5_6_7/Fig_6b.pdf]

Rotate
Zoom
Pan

The 3'-endo 2'dCTP fitted into the omit map (3σ) of the RNAP-2'dCTP complex. The 3'OH can form a hydrogen bond with only one RNAP atom (magenta line, interatomic distance 3.1 Å).
