# Supplementary material for: The mechanism of the nucleo-sugar selection by multi-subunit RNA polymerases
Source: Nat Commun. 2021 Feb 4;12:796. doi: 10.1038/s41467-021-21005-w (PMC7862312; doi:10.1038/s41467-021-21005-w)

The mechanism of the nucleo-sugar selection by multi-subunit RNA polymerases
Janne J. Mäkinen[1], Yeonoh Shin[2], Eeva Vieras[1], Pasi Virta[3], Mikko Metsä-Ketelä[1], Katsuhiko S. Murakami[2]*, Georgiy A. Belogurov[1]*

This document contains low resolution and low bit depth annotated previews. Original, unprocessed 16-bit scans are supplied as separate files with the corresponding names.

Fig. 4

J2.TIF

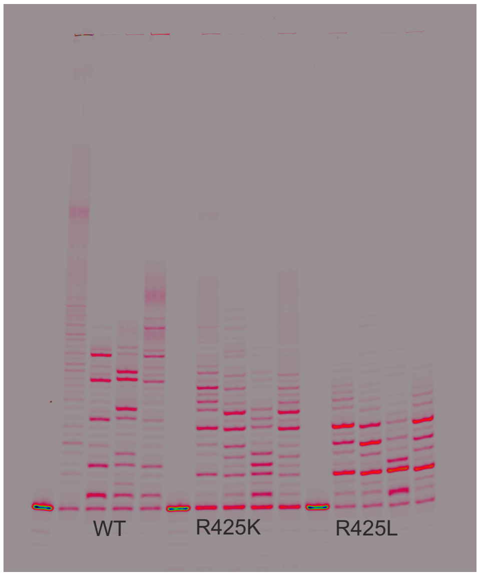

J1.TIF

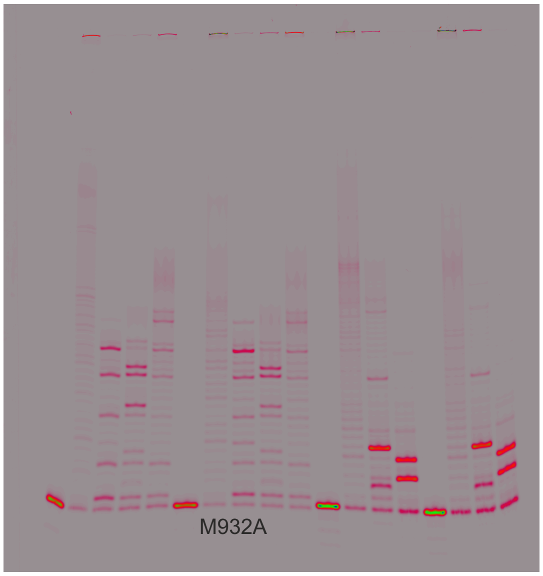

J3.TIF

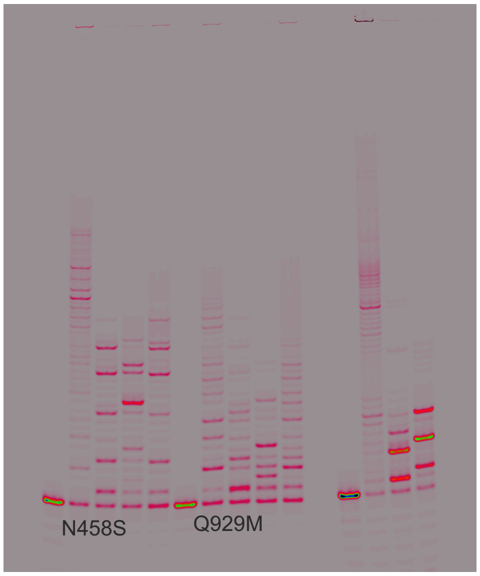

The mechanism of the nucleo-sugar selection by multi-subunit RNA polymerases

Janne J. Mäkinen[1], Yeonoh Shin[2], Eeva Vieras[1], Pasi Virta[3], Mikko Metsä-Ketelä[1], Katsuhiko S. Murakami[2]*, Georgiy A. Belogurov[1]*

This document contains low resolution and low bit depth annotated previews. Original, unprocessed 16-bit scans are supplied as separate files with the corresponding names.

Supplementary Fig. 4

E20.TIF

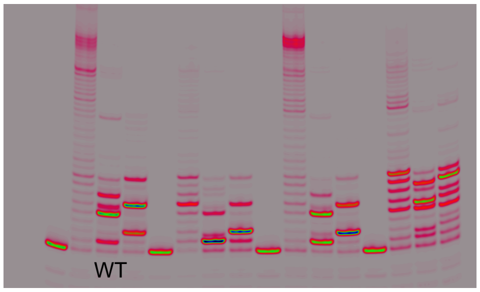

E27.TIF

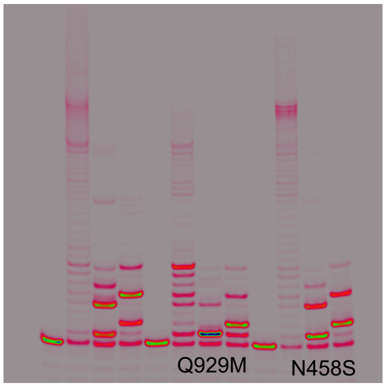

V2.TIF

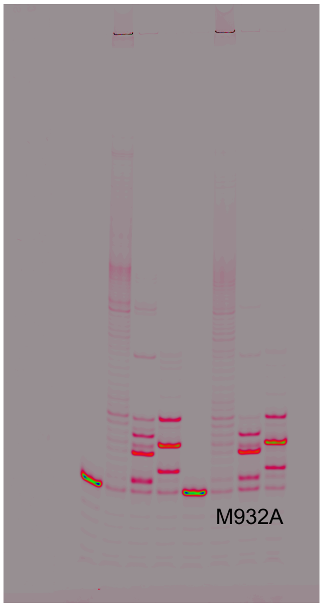

E22.TIF

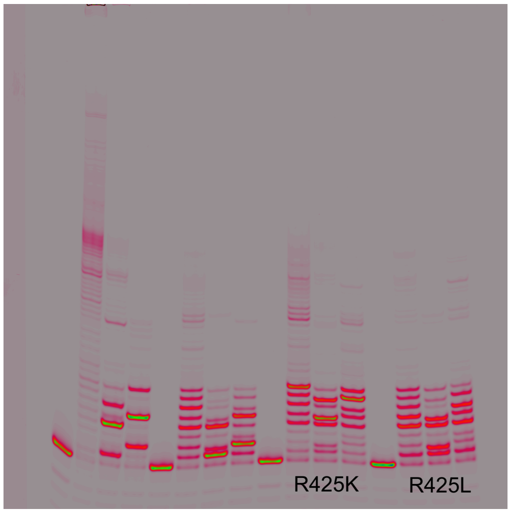

The mechanism of the nucleo-sugar selection by multi-subunit RNA polymerases

Janne J. Mäkinen[1], Yeonoh Shin[2], Eeva Vieras[1], Pasi Virta[3], Mikko Metsä-Ketelä[1], Katsuhiko S. Murakami[2]*, Georgiy A. Belogurov[1]*

This document contains low resolution and low bit depth annotated previews. Original, unprocessed 16-bit scans are supplied as separate files with the corresponding names.

Supplementary Fig. 6

E36.TIF

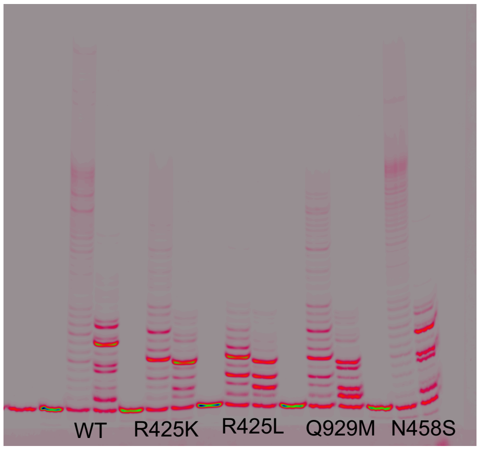

E37.TIF

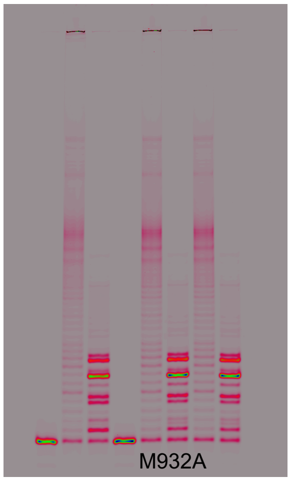

Supplement: Supplementary file 4 — Source Data [file 41467_2021_21005_MOESM4_ESM.zip › Transcriptiopn_gels_Fig_4_S4_S6/Gel_annotations.pdf]
