# Supplementary figures and images for: The mechanism of the nucleo-sugar selection by multi-subunit RNA polymerases
Source: Nat Commun. 2021 Feb 4;12:796. doi: 10.1038/s41467-021-21005-w (PMC7862312; doi:10.1038/s41467-021-21005-w)

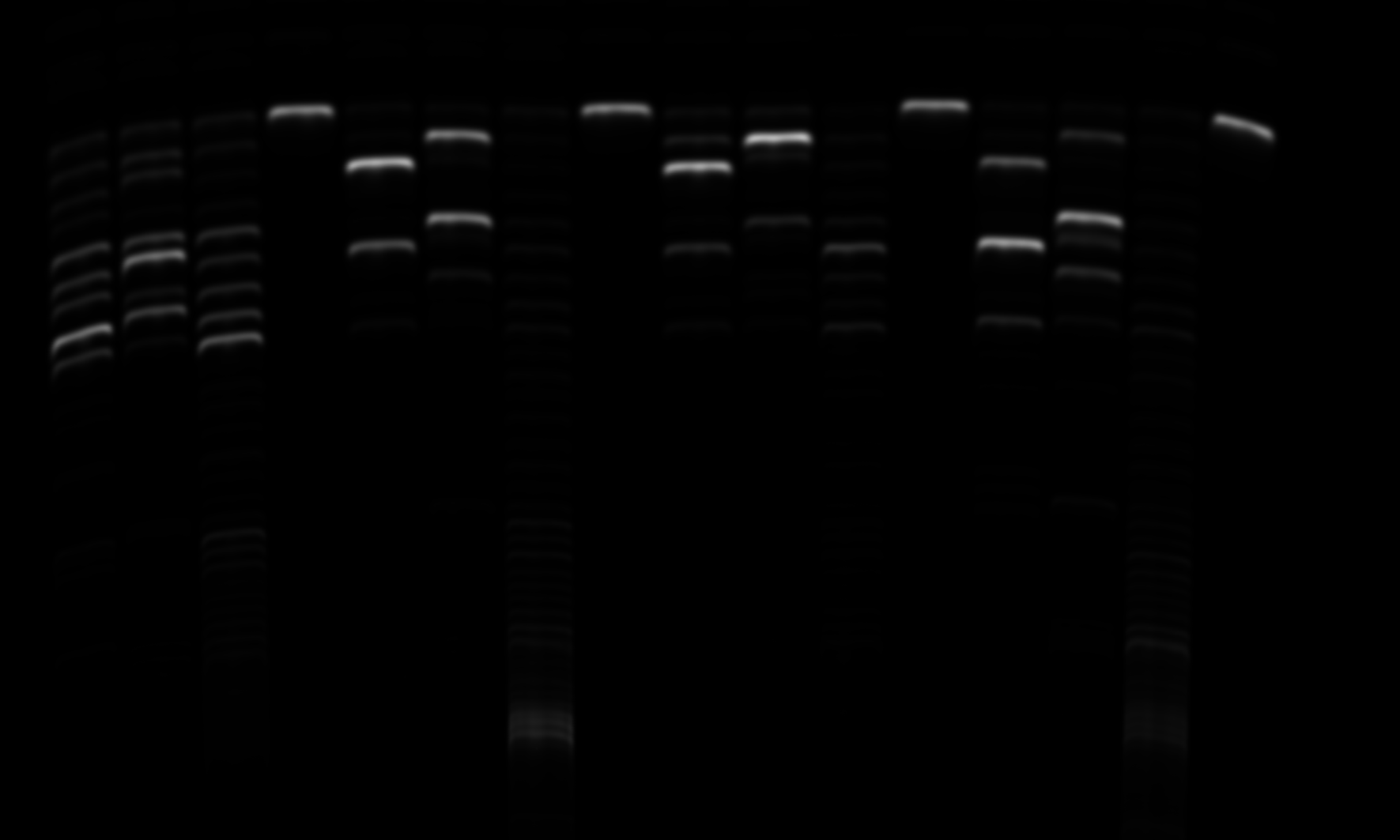

Supplement: Supplementary file 4 — Source Data [file 41467_2021_21005_MOESM4_ESM.zip › Transcriptiopn_gels_Fig_4_S4_S6/E20.TIF]

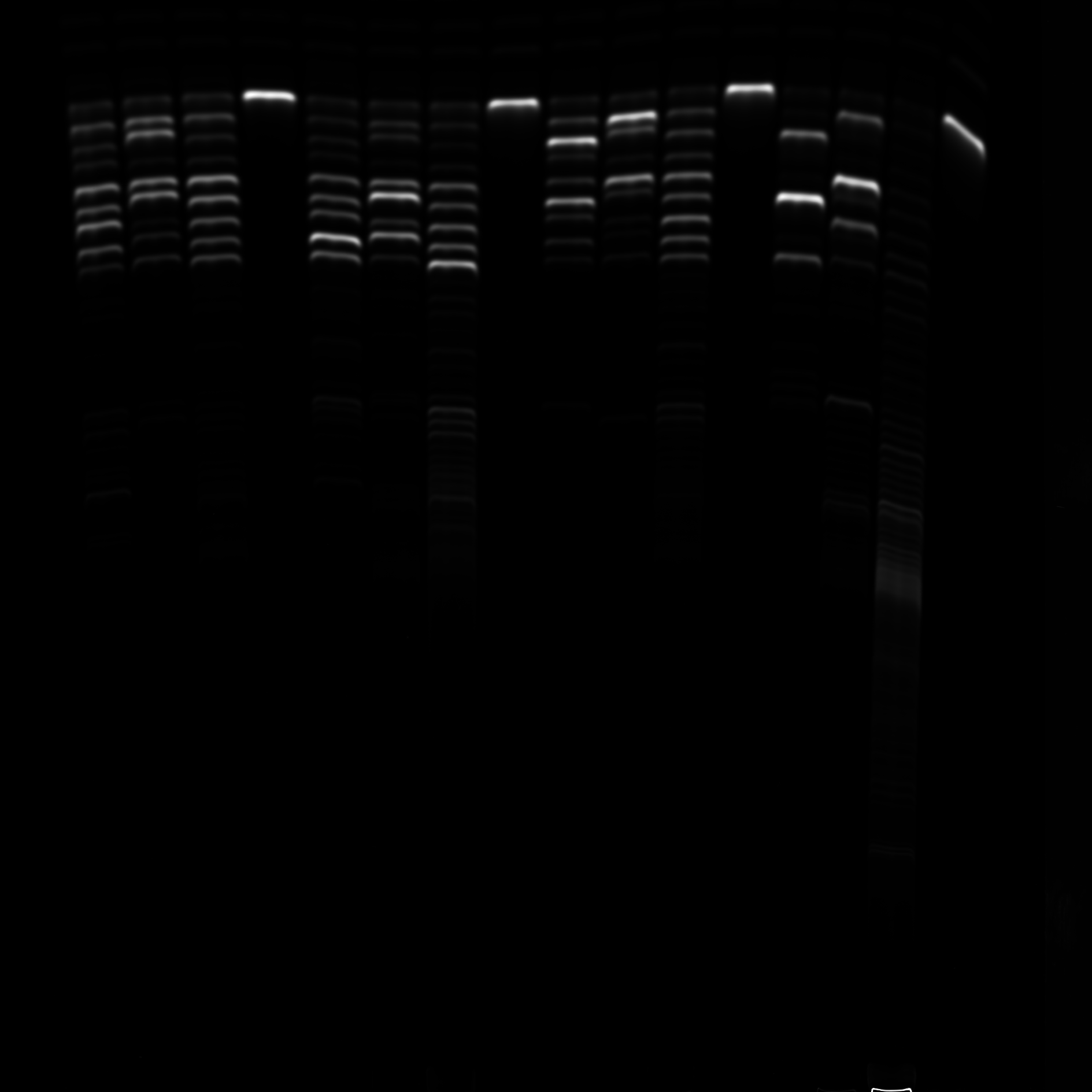

Supplement: Supplementary file 4 — Source Data [file 41467_2021_21005_MOESM4_ESM.zip › Transcriptiopn_gels_Fig_4_S4_S6/E22.TIF]

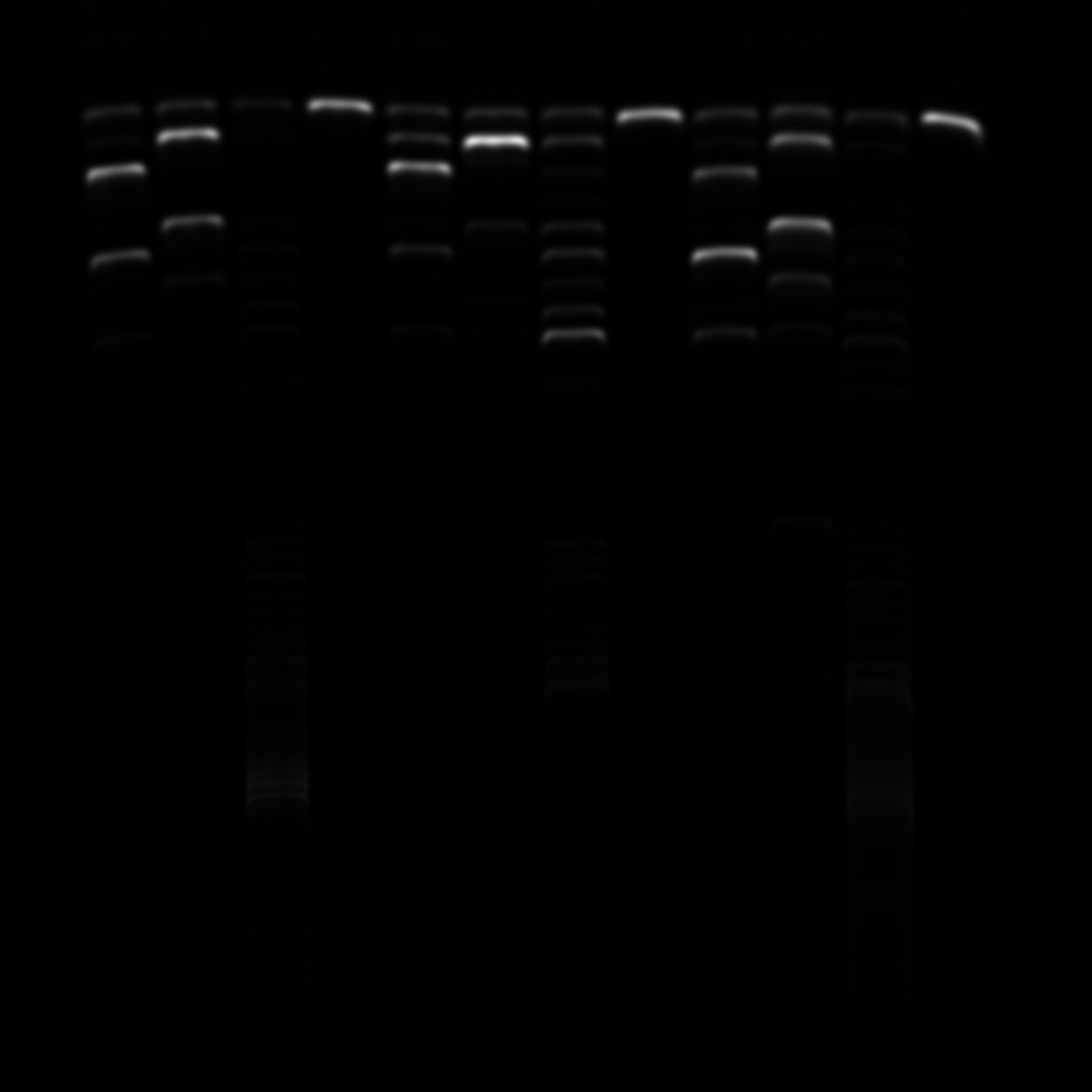

Supplement: Supplementary file 4 — Source Data [file 41467_2021_21005_MOESM4_ESM.zip › Transcriptiopn_gels_Fig_4_S4_S6/E27.TIF]

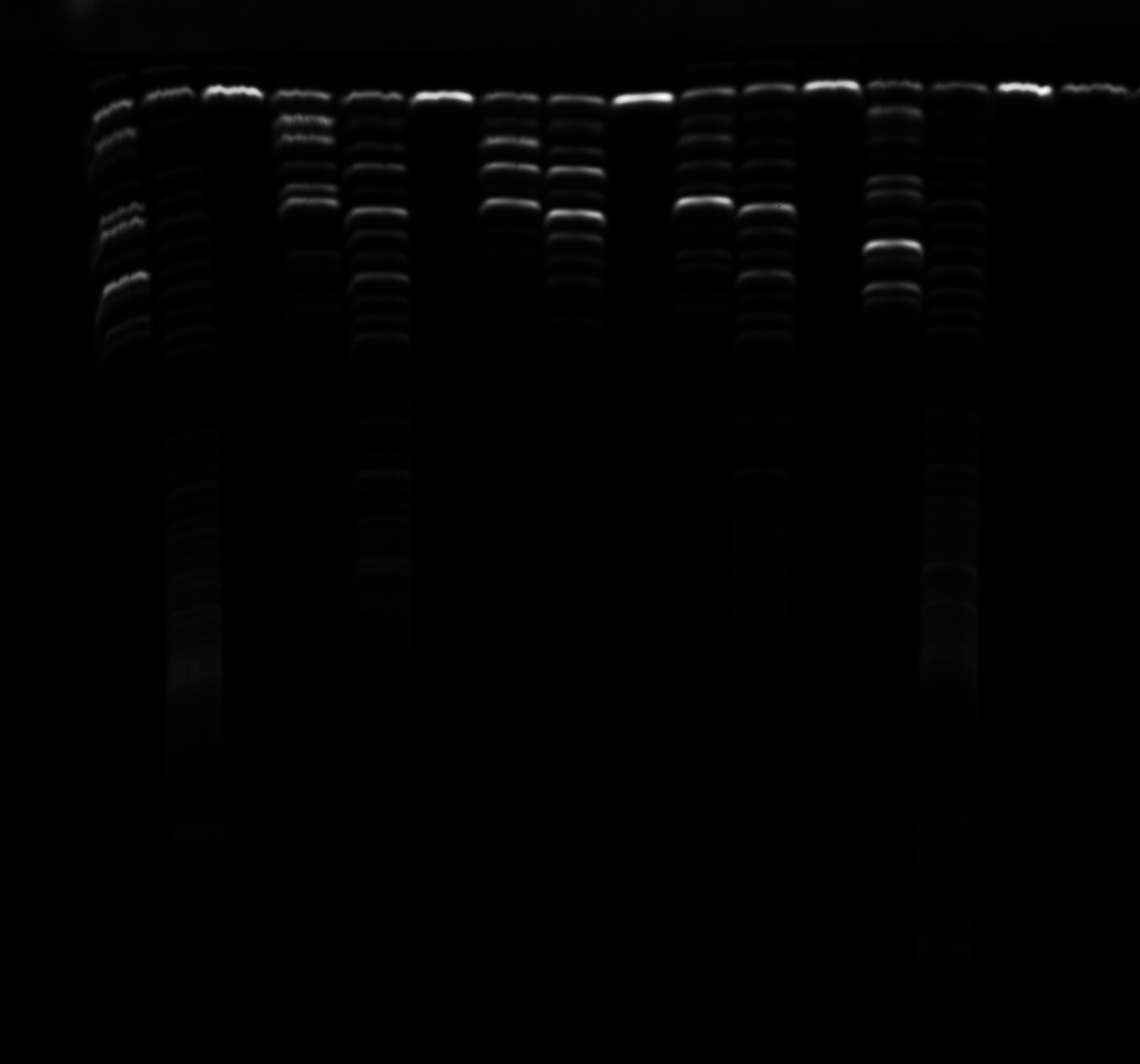

Supplement: Supplementary file 4 — Source Data [file 41467_2021_21005_MOESM4_ESM.zip › Transcriptiopn_gels_Fig_4_S4_S6/E36.TIF]

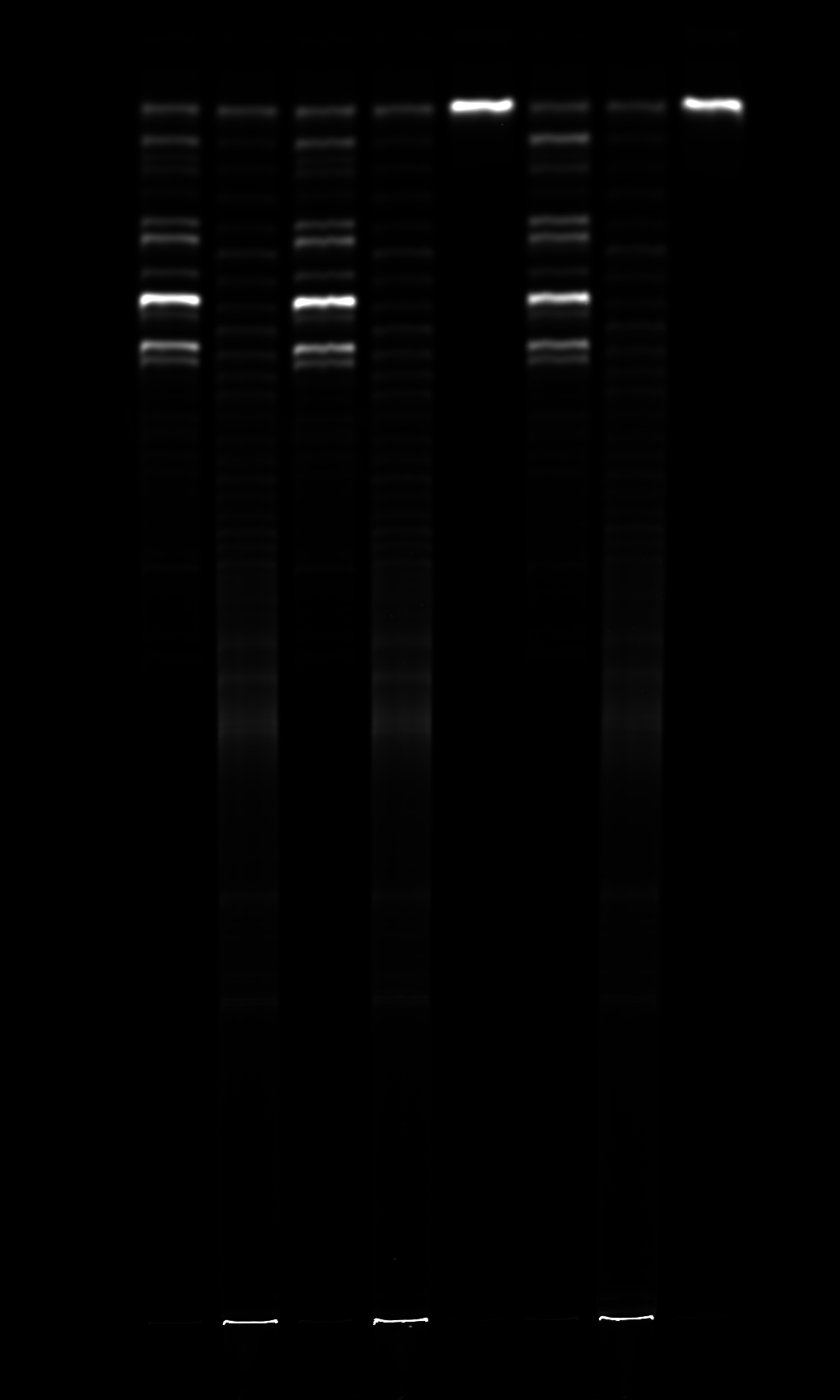

Supplement: Supplementary file 4 — Source Data [file 41467_2021_21005_MOESM4_ESM.zip › Transcriptiopn_gels_Fig_4_S4_S6/E37.TIF]

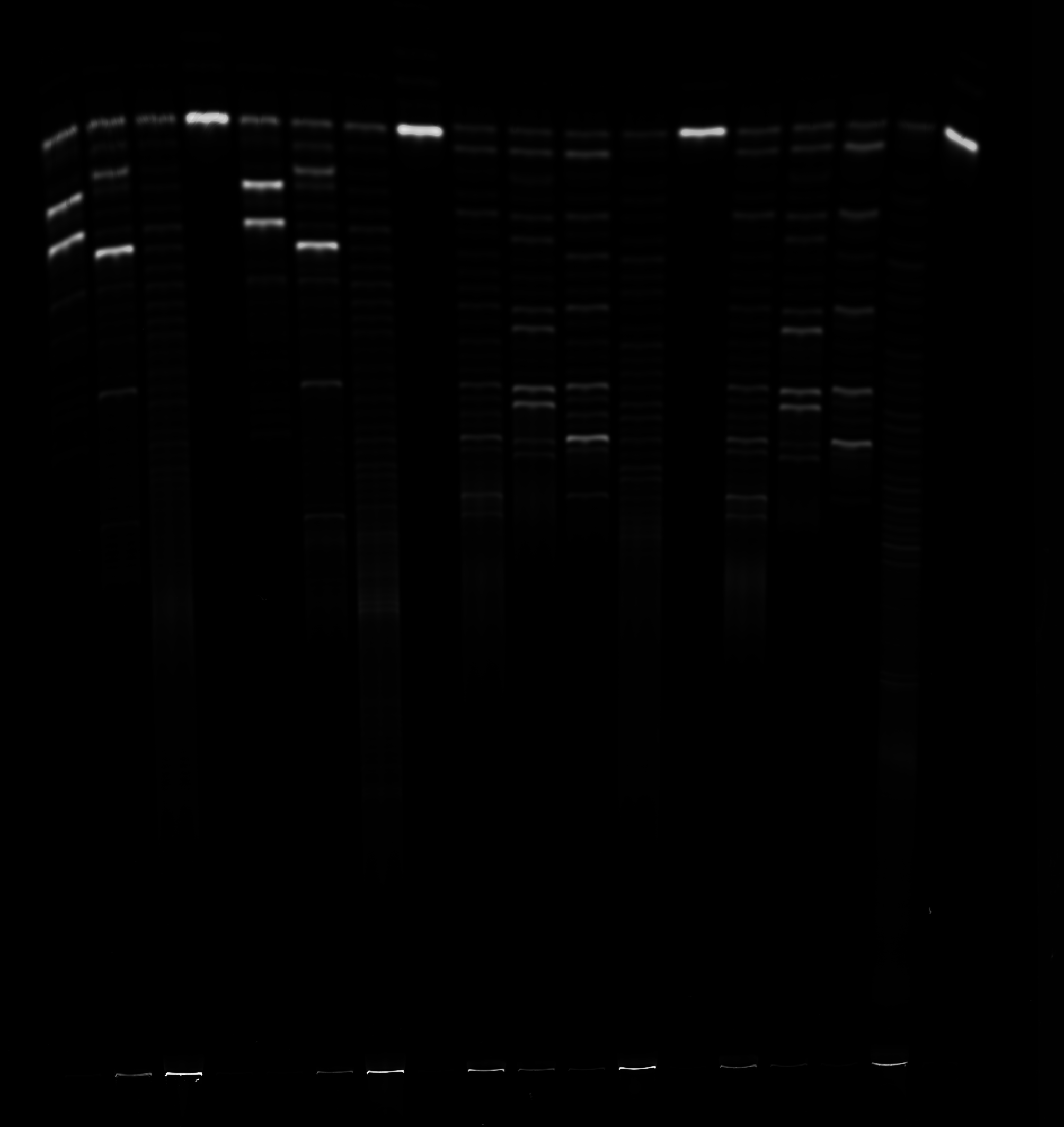

Supplement: Supplementary file 4 — Source Data [file 41467_2021_21005_MOESM4_ESM.zip › Transcriptiopn_gels_Fig_4_S4_S6/J1.TIF]

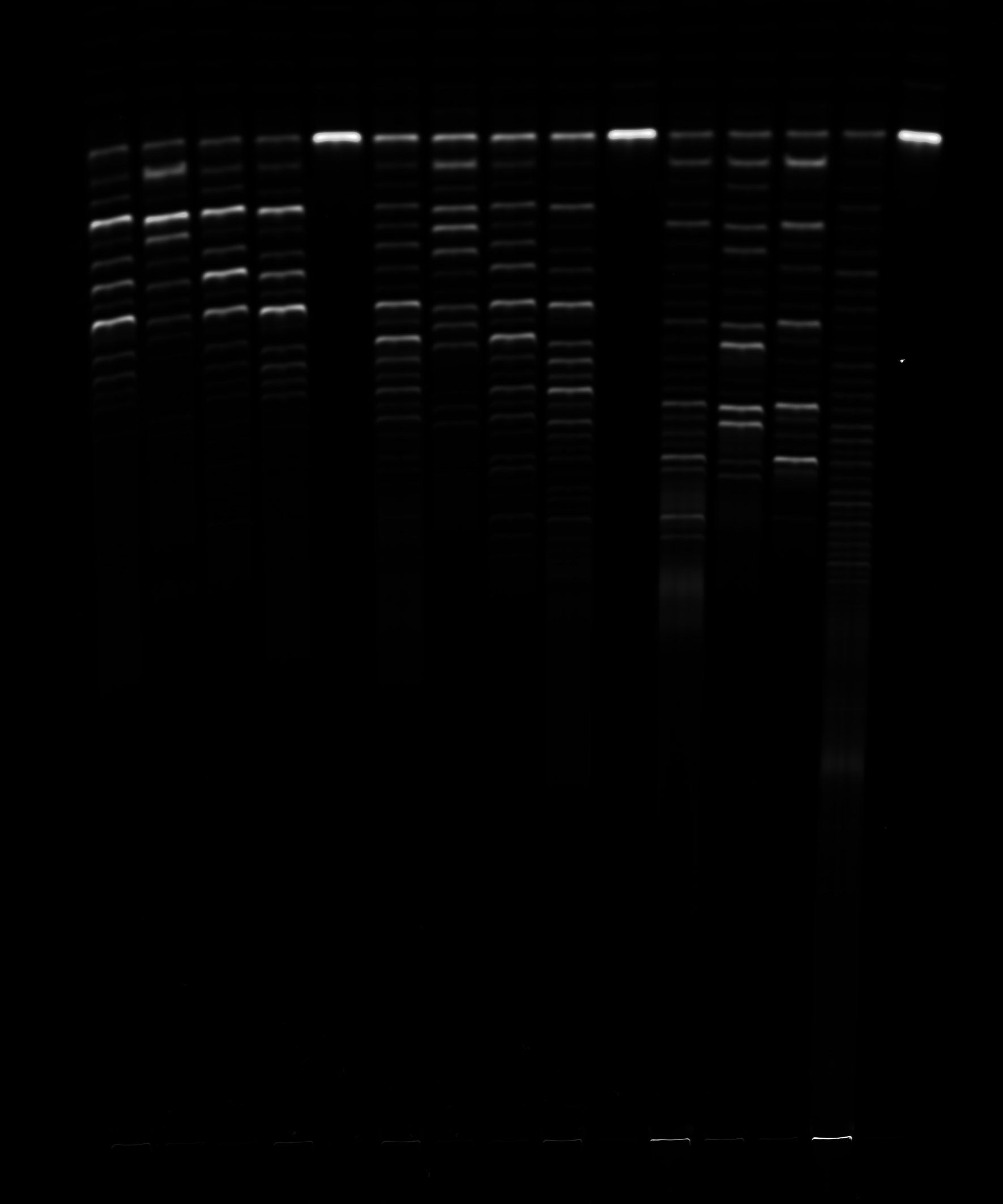

Supplement: Supplementary file 4 — Source Data [file 41467_2021_21005_MOESM4_ESM.zip › Transcriptiopn_gels_Fig_4_S4_S6/J2.TIF]

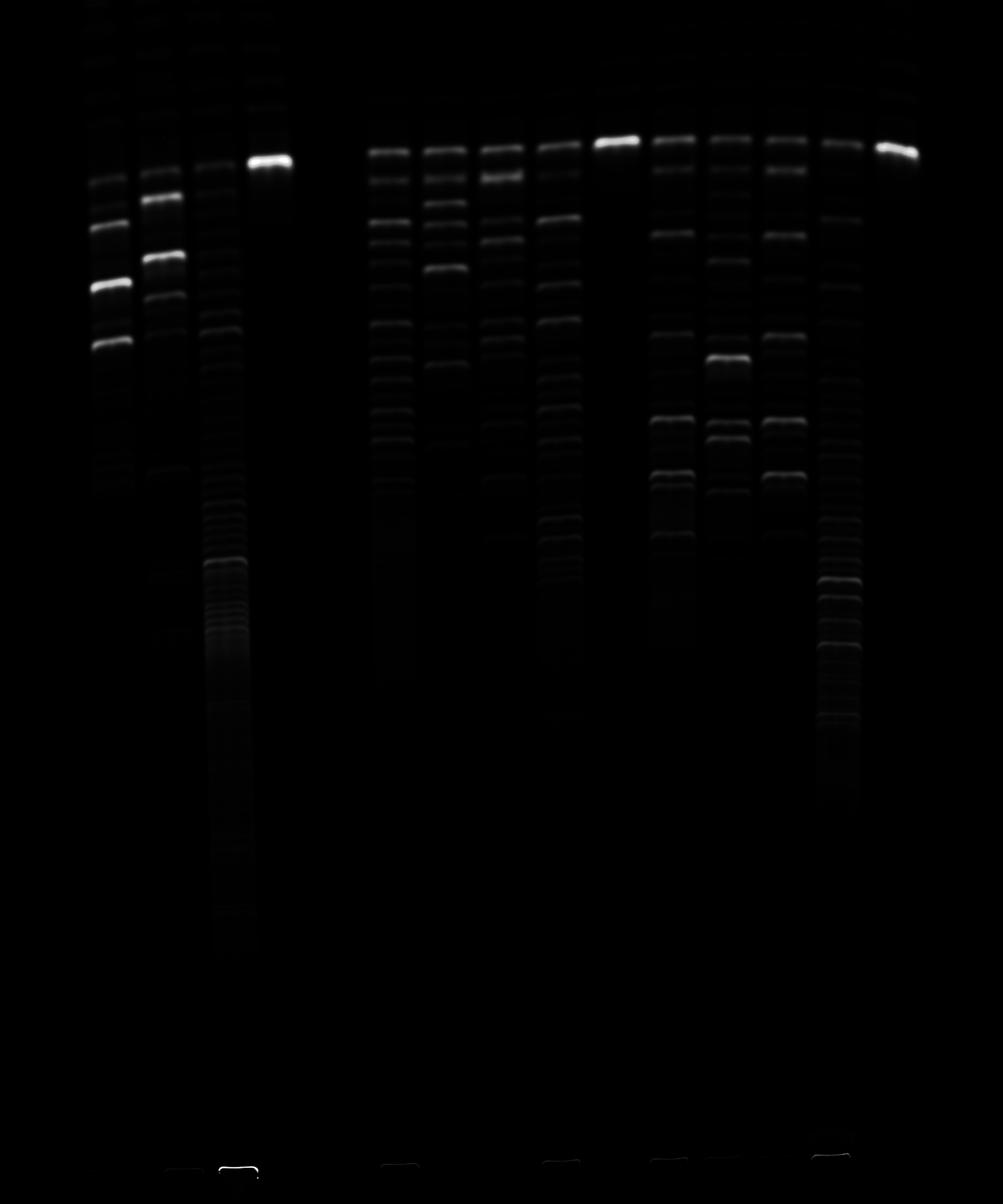

Supplement: Supplementary file 4 — Source Data [file 41467_2021_21005_MOESM4_ESM.zip › Transcriptiopn_gels_Fig_4_S4_S6/J3.TIF]

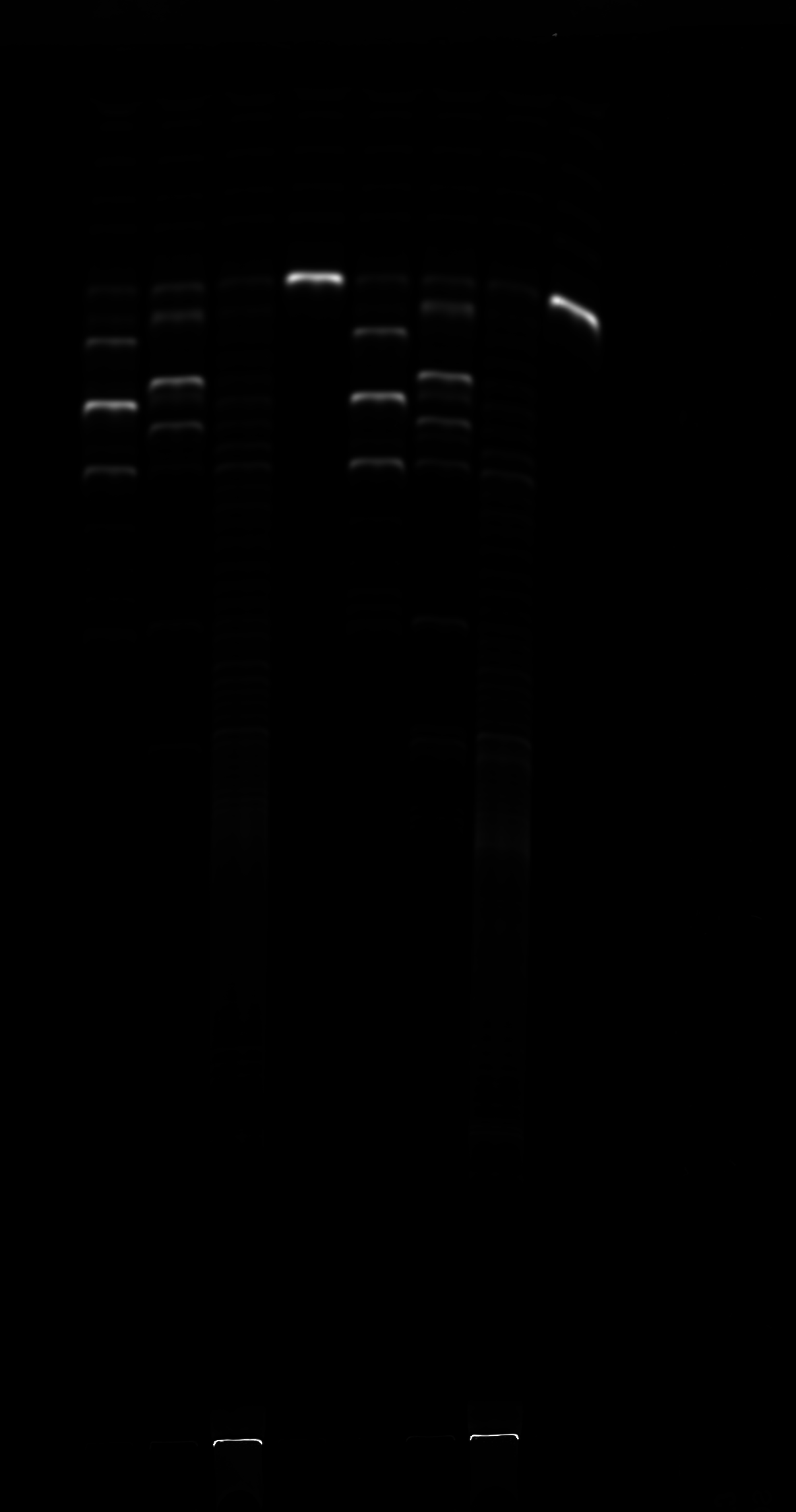

Supplement: Supplementary file 4 — Source Data [file 41467_2021_21005_MOESM4_ESM.zip › Transcriptiopn_gels_Fig_4_S4_S6/V2.TIF]
