# Supplementary material for: The mechanism of the nucleo-sugar selection by multi-subunit RNA polymerases
Source: Nat Commun. 2021 Feb 4;12:796. doi: 10.1038/s41467-021-21005-w (PMC7862312; doi:10.1038/s41467-021-21005-w)
Supplement: Supplementary file 2 — Peer Review File [file 41467_2021_21005_MOESM2_ESM.pdf]

Reviewer #1 (Remarks to the Author):

The manuscript "The mechanism of the nucleo-sugar selection by multi-subunit RNA polymerases," by Mäkinen et al presents an interesting study of a fundamental question in RNA polymerase function. The combination of structural studies with biophysical ones allows direct testing of observed interactions. In general, this a well constructed study that should be of interest across the broad field of nucleic acid polymerases.

In the introduction (page 2), regarding the sentence: "These steric gate residues, typically Gln/Glu in A-family DNAPs and Tyr/Phe in Y-and B-family DNAPs, create a stacking interaction with the deoxyribose moiety of an incoming 2'dNTP," what do the authors mean by a "stacking" interaction with a sugar?
More importantly, for the single subunit polymerases, elegant studies have shown that removal of the tyrosyl hydroxyl does not reduce the rate of rNMP incorporation, but rather, increases the rate of dNMP incorporation. Thus the sentence in the current manuscript "Tyr selectively facilitates the binding of NTPs by forming a hydrogen bond with the 2'OH group of the NTP ribose" is in disagreement with this literature, or is at least too simplistic. A follow-on caveat sentence in the current manuscript attempts to address this, but does not alter this disagreement. Some revision of this section is in order.
This is interesting in that the current manuscript shows similar effects for the β'R425K mutant (and perhaps related, partial effects for β'Gln929). While the details are still not clear, the correspondence in behavior is intriguing.

Minor issues:

Throughout the manuscript, C'4 should be corrected to C4'
In the introduction, the manuscript states "A-form geometry because 2'OH groups in the RNA clash with the phosphate linkages in the B-form configuration." This ignores the "gauche/pseudo-rotation model" for such effects - has the latter been disproven?

Reviewer #2 (Remarks to the Author):

The paper entitled, "The mechanism of the nucleo-sugar selection by multi-subunit RNA polymerases" from the Belogurov and Murakami labs is a thorough analysis of the nucleotide selection mechanism for NTPs vs dNTPs by RNAPs. Overall, the paper is well written and of interest to the readers of Nature Communications. The authors show that multi-subunit RNAPs use a conserved Arg residue to selectively inhibit incorporation of 2'dNTPs. This is in contrast to single subunit RNAPs that utilize a conserved Tyr residue. Furthermore, structural snapshots show the trigger loop is unfolded in the presence of dNTPs and not NTPs.

The major concern for this reviewer is the ability to determine the sugar conformation of the dNTPs in the crystal structures, which is a major conclusion of the paper. At the given resolution ~3 angstroms and with the presented density figures, this reviewer is not very convinced of the ability to unequivocally determine the sugar pucker of the dNTP in panel 4d vs 4f. This should be addressed in the manuscript to strengthen their conclusions through more structural analysis (e.g. difference/polder maps to get clear density or showing multiple types of sugar fits) or additional experimental evidence (possibly already published in prior studies). It would help strengthen the paper if this data analysis was more convincing.

Minor comments are listed below:

1. The bottom panel of Figure 1C should be its own panel (e.g. 1D) to help with flow and clarity.

2. The introduction is a bit long. The two paragraphs about the TL could be merged into one and shortened.
3. The opening sentence in the results section states "…we performed time-resolved studied…". It is not clear what the authors mean by time-resolved studies in this statement as written. They should clarify this term for the reader.
4. Supplementary Fig. 1 should be merged with the main text Figure 2. This is a very important figure to understanding the assay. Furthermore, the bar graphs are not the best way to show the data in Fig 2. This reviewer does acknowledge Table 1 lists all the values, but as presented the data is now spread out across multiple figures and a table.
5. Does an R425A mutation have the same impact as the R425K mutation that maintains the charge?
6. In general, the supplemental information section is too large in comparison to the main figures of the text. It would be nice to have more of the supplemental data in the main part of the text, if space would allow.
7. As a point of clarification, did the authors use natural dCTP in the crystallization studies and if so how did they prevent catalysis in the crystal from occurring over time.
8. The discussion should be substantially shortened by removing aspects that are already discussed in the results section and focusing it for the reader.

Reviewer #1 (Remarks to the Author):

The manuscript “The mechanism of the nucleo-sugar selection by multi-subunit RNA polymerases,” by Mäkinen et al presents an interesting study of a fundamental question in RNA polymerase function. The combination of structural studies with biophysical ones allows direct testing of observed interactions. In general, this is a well constructed study that should be of interest across the broad field of nucleic acid polymerases.

Response: We thank the reviewer for the positive evaluation of the scientific quality and significance of our study.

In the introduction (page 2), regarding the sentence: “These steric gate residues, typically Gln/Glu in A-family DNAPs and Tyr/Phe in Y-and B-family DNAPs, create a stacking interaction with the deoxyribose moiety of an incoming 2’dNTP,” what do the authors mean by a “stacking” interaction with a sugar?

Response: We rephrased the relevant sentence as follows: “The steric gate residue, typically Gln/Glu in A-family DNAPs and Tyr/Phe in Y-and B-family DNAPs, stretches along the α-face of the

deoxyribose moiety of an incoming 2'dNTP and forms a hydrogen bond between the backbone amide group and the 3'-OH group of 2'dNTP (Fig. 1d)."

More importantly, for the single subunit polymerases, elegant studies have shown that removal of the tyrosyl hydroxyl does not reduce the rate of rNMP incorporation, but rather, increases the rate of dNMP incorporation. Thus the sentence in the current manuscript "Tyr selectively facilitates the binding of NTPs by forming a hydrogen bond with the 2'OH group of the NTP ribose" is in disagreement with this literature, or is at least too simplistic. A follow-on caveat sentence in the current manuscript attempts to address this, but does not alter this disagreement. Some revision of this section is in order.

Response: We rephrased the relevant sentences as follows: "Tyr forms a hydrogen bond with the 2'OH group of the NTP ribose (Fig. 1c) but mediates the selectivity by inhibiting the binding and incorporation of 2'dNTPs. It is hypothesized that the formation of the Tyr - 2'OH hydrogen bond upon the binding of NTPs counteracts an inhibitory interaction of the Tyr with another residue or a water molecule."

This is interesting in that the current manuscript shows similar effects for the 13'R425K mutant (and perhaps related, partial effects for 13'Gln929). While the details are still not clear, the correspondence in behavior is intriguing.

Response: There is indeed a very intriguing correlation in terms of the effect on kcat: both mutations increase kcat for 2'dNTPs without significantly affecting kcat for NTPs. However, the altered T7 RNAP maintains a low Kd for NTPs, whereas R425K in *E. coli* RNAP increases Kd hundred-fold. The unchanged Kd for NTPs in the mutant T7 RNAP is perplexing and suggests a complex mechanism involving inhibitory interaction of the discriminating Tyr with water or other residues (Biochemistry 1997; 36(27):8231-42). In contrast, the discriminating action of R425 can be explained solely by alternative interactions with sugar moieties of ribo- and deoxyribonucleoside substrates, as we report here. Another important difference is that the discriminating Tyr in T7 and N4 RNAPs undergoes a large motion (as part of a mobile O-helix; Proc. Natl. Acad. Sci. U S A. 2011;108(9):356671) during NTP binding that complicates the interpretation of its effects. In contrast, R425 belongs to the "stationary" active site cavity and, as we report here, maintains the same conformation during the binding and incorporation of both ribo- and deoxyribonucleoside substrates.

Minor issues:

Throughout the manuscript, C'4 should be corrected to C4'

Response: Implemented.

In the introduction, the manuscript states "A-form geometry because 2'OH groups in the RNA clash with the phosphate linkages in the B-form configuration." This ignores the "gauche/pseudo-rotation model" for such effects - has the latter been disproven?

Response: We reformulated the sentence as follows: "Hybrid duplexes between the RNA and DNA transiently form during transcription and adopt an A-form geometry because conformational preferences of the RNA strand outweigh those of the more flexible DNA strand."

With respect to the "gauche/pseudo-rotation model", it is well established that electronegative substituents at C2' favor the 3'-endo conformer of the nucleo-sugar (a specific case of the gauche effect). Similarly, it is commonly accepted that the interconversion of 2'-endo and 3'-endo nucleo-sugars occurs by pseudorotation via O4'-endo transition state rather than via the inversion involving

a planar intermediate. In general, the pseudorotation model is widely employed to describe the free energy landscape of nucleo-sugar conformations. However, in our view, the pseudorotation model does not offer a simple explanation for the inability of RNA to adopt a B-form helix. Thus, monomeric ribonucleosides and their phosphorylated derivatives still prefer 2'-endo conformation in solution despite featuring 2'OH (J. Am. Chem. Soc. 2012; 134(8):3691-4). The strong preference for the 3'-endo conformation and A-form helix is only characteristic for the 3'-5' linked RNA. This preference is a result of a superimposition of several gauche effects within the ribose ring, effects of phosphate linkages, stacking of nucleobases and conformational restrictions imposed by Watson-Crick pairing. The observation that ribose moieties adopt 2'-endo conformations in the A-form helix formed by the 2'-5' linked RNA (J Am Chem Soc. 2014; 136(7):2858-65) further highlights the complexity of the situation.

We attempted to provide a simple explanation for the inability of RNA to adopt a B-form helix within the framework of the pseudorotation model, but ultimately decided against elaborating on the matter in the manuscript text. After all, the purpose of the paragraph in question was merely to introduce the conformational diversity of biologically relevant nucleic acids and individual building blocks.

Reviewer #2 (Remarks to the Author):

The paper entitled, "The mechanism of the nucleo-sugar selection by multi-subunit RNA polymerases" from the Belogurov and Murakami labs is a thorough analysis of the nucleotide selection mechanism for NTPs vs dNTPs by RNAPs. Overall, the paper is well written and of interest to the readers of Nature Communications. The authors show that multi-subunit RNAPs use a conserved Arg residue to selectively inhibit incorporation of 2'dNTPs. This is in contrast to single subunit RNAPs that utilize a conserved Tyr residue. Furthermore, structural snapshots show the trigger loop is unfolded in the presence of dNTPs and not NTPs.

Response: We thank the reviewer for the positive evaluation of the scientific quality and significance of our study.

The major concern for this reviewer is the ability to determine the sugar conformation of the dNTPs in the crystal structures, which is a major conclusion of the paper. At the given resolution ~3 angstroms and with the presented density figures, this reviewer is not very convinced of the ability to unequivocally determine the sugar pucker of the dNTP in panel 4d vs 4f. This should be addressed in the manuscript to strengthen their conclusions through more structural analysis (e.g. difference/polder maps to get clear density or showing multiple types of sugar fits) or additional experimental evidence (possibly already published in prior studies). It would help strengthen the paper if this data analysis was more convincing.

Response: We agree with the reviewer that the resolution of our X-ray crystal structures (as well as all structures of multi-subunit RNAPs determined to date) is insufficient to unambiguously determine the conformation of the nucleo-sugar from the electron density alone. However, we argue that the 2'-endo conformer is a better interpretation of the electron density when several additional lines of evidence are considered. We further fulfill the reviewer's suggestion to explicitly discuss these additional considerations and perform alternative sugar fits:

First, the electron density maps rule out significant rearrangements of amino acid side chains in the active site cavity upon the binding of 2'dCTP. In particular, there is a well-resolved density for Arg425 sidechain that is localized similarly in structures with CMPCPP, 2'dCTP and 3'dCTP. In all those structures, Arg425 bridges 2'OH of the RNA primer and a main chain carbonyl of Tyr(Phe)457

(Supplementary Fig. 8c). The invariability of side chain conformations in the active site cavity narrows down the explanation for the inertness of 2’dCTP to a difference in the conformation of the trigger loop (TL, see below), a difference in the conformation of the sugar moiety, or both.

Second, the TL is unfolded in structures with 2’dCTP and 3’dCTP but is folded up to Met932 in most structures with CMPCPP. The latter observation suggests that (i) the interaction between 3’OH and Gln929 is important for stabilizing the TL folding, and (ii) 3’OH is unavailable for interaction with Gln929 in the 2’dCTP structure. The latter is an expected consequence of the 2’-endo binding pose (Fig. 5d). In contrast, the 3’-endo conformer is expected to position the 3’OH to interact with the TL (Fig. 5c) similarly to CMPCPP (Fig. 5b). We now rephrased the Results section to state more explicitly that the unfolded TL suggests that 2’dCTP binds in the 2’-endo conformation.

Third, we performed alternative sugar fits into the omit map and illustrated the result (Fig. 6). For this comparison we extracted the 2’-endo and 3’-endo conformers from high resolution structures and fitted them into the omit map by rotating bonds but preserving bond lengths and angles and avoiding treating ligands with force-fields of any kind. While the bulk of either conformer can be accommodated withing the omit map, the 3’OH group is partially (2’-endo) or completely (3’-endo) unrepresented by the electron density. Strikingly, the 3’OH of the 2’-endo conformer is positioned to form up to three hydrogen bonds with RNAP atoms, whereas the 3’OH of the 3’-endo conformer can only form a single hydrogen bond. These observations suggest that 2’-endo conformer interacts more favorably with RNAP and the 2’-endo binding pose is a better interpretation of the electron density. Noteworthy, the 2’-endo binding pose is also robustly recovered (Fig. 5d, Supplementary table 3) by a reputable docking algorithm (Autodock Vina) that was trained on a large set of high-resolution X-ray crystal structures and therefore has an objective predicting power. We now explicitly discuss these reasonings in the manuscript and further reinforce our inferences by considering the intrinsic conformational preferences of 2’dNTPs.

Minor comments are listed below:

1. The bottom panel of Figure 1C should be its own panel (e.g. 1D) to help with flow and clarity. Response: implemented

2. The introduction is a bit long. The two paragraphs about the TL could be merged into one and shortened.

Response: implemented.

3. The opening sentence in the results section states “...we performed time-resolved studied...”. It is not clear what the authors mean by time-resolved studies in this statement as written. They should clarify this term for the reader.

Response: we replaced “studied” [sic] with “measurements”.

4. Supplementary Fig. 1 should be merged with the main text Figure 2. This is a very important figure to understanding the assay. Furthermore, the bar graphs are not the best way to show the data in Fig 2. This reviewer does acknowledge Table 1 lists all the values, but as presented the data is now spread out across multiple figures and a table.

Response: Our data are multidimensional: several substrates, several RNAPs, two analysis routines, multiple parameters for each substrate-RNAP-analysis-routine combination. To substantiate our inferences, we drew several projections of our multidimensional data that inevitably lead to some degree of fragmentation in data presentation. To address the reviewers queries we moved

Supplementary Figure 1 (now Fig. 2) and Supplementary Table 4 (now Table 2) into the main text. We further suggest that Figure 3 and Tables 1-2 should be placed together on the same page to minimize the fragmentation of the data presentation. We understood the reviewer's concern about Figure 3 (former Fig. 2) and experimented with alternative presentations, but ultimately decided to keep bar graphs and a linear Y-scale. Figure 3 combines data obtained using two different analysis routines: it would be difficult to merge these data in one table or compare them across two tables. We further argue that fold differences indicated below the graphs in Figure 3 will allow readers to precisely grasp the magnitude of the effects, despite several small values being illegible as bars.

5. Does an R425A mutation have the same impact as the R425K mutation that maintains the charge?

Response: In Figure 4a, Supplementary Figure 3 and 6 we show that R425L is markedly defective in processive transcription but discriminates poorly against 2'dNTPs similarly to R425K. We included R425L data to demonstrate that the impaired discrimination against 2'dNTPs by R425K is due to the absence of Arg rather than the presence of Lys. R425L removes the charge and fills the volume occupied by an aliphatic part of the Arg425 side chain. In contrast, R425A would create a large void in the critical area near the 3' end of the nascent RNA. In general, RNAPs with substitutions of R425 express poorly and are very difficult to purify. Given possible indirect effects of an Ala substitution, we did not test if R425A can be expressed and purified.

6. In general, the supplemental information section is too large in comparison to the main figures of the text. It would be nice to have more of the supplemental data in the main part of the text, if space would allow.

Response: To address the reviewer's query we moved Supplementary Figures 1 (now Fig. 2), Supplementary Figure X (now Fig. 5) and Supplementary Table 4 (now Table 2) from the supplementary information to the main text.

7. As a point of clarification, did the authors use natural dCTP in the crystallization studies and if so how did they prevent catalysis in the crystal from occurring over time.

Response: We soaked crystals with natural 2'dCTP and 3'dCTP for 0.5 min that was sufficient for the binding but insufficient for the incorporation. The bimolecular reaction of substrate binding is very fast at saturating concentrations of 2'dCTP (4 mM) and 3'dCTP (4 mM) even in crystals. At the same time, the rate of the phosphodiester bond formation by the thermophilic RNAP *in crystallo* is substantially slower than the one in solution as we reported in the recent paper (Nucleic Acids Res. 2020; 48(4): 2144-2155). In the revised version, we indicated the soaking time in the Methods section. We also added a statement "Pre-catalytic complexes in (c) and (d) were trapped due to the low reactivity of deoxyribonucleoside substrates and the slow catalysis by thermophilic RNAP in crystallo [52]" to Figure 7 legend.

8. The discussion should be substantially shortened by removing aspects that are already discussed in the results section and focusing it for the reader.

Response: we significantly shortened the discussion and reduced the redundancy between Results and Discussion sections.

Reviewer #2 (Remarks to the Author):

The authors addressed my concerns in full.
